# Assessment of the chemical and genetic variability among accessions of *Cicerbita alpina* (L.) Wallr., an alpine plant with anthelmintic properties

**DOI:** 10.3389/fpls.2023.1269613

**Published:** 2023-11-20

**Authors:** Eftychia Martinidou, Luisa Palmieri, Maddalena Sordo, Domenico Masuero, Maria Ourda, Luca Delucchi, Pietro Fusani, Veronika Tremml, Ioanna Poulopoulou, Matthias Gauly, Mark J. Horgan, Bianka Siewert, Hermann Stuppner, Stefan Martens

**Affiliations:** ^1^ Research and Innovation Center, Edmund Mach Foundation, San Michele all’Adige, Trento, Italy; ^2^ Centro di Ricerca Foreste e Legno, Consiglio per la Ricerca in Agricoltura e l’Analisi dell’Economia Agraria, Trento, Italy; ^3^ Department of Pharmaceutical and Medicinal Chemistry, Institute of Pharmacy, Paracelsus Medical University Salzburg, Salzburg, Austria; ^4^ Faculty of Agricultural, Environmental and Food Sciences, Free University of Bolzano, Bolzano, Italy; ^5^ Institute of Pharmacy/Pharmacognosy, Center for Chemistry and Biomedicine, University of Innsbruck, Innsbruck, Austria

**Keywords:** anthelmintic, biodiversity, sesquiterpene lactones, genotyping, chemical diversity, *Cicerbita alpina*, ethnoveterinary, medicinal and aromatic plants

## Abstract

*Cicerbita alpina* (L.) Wallr, is a perennial alpine plant and a member of the *Asteraceae* family, typically found at altitudes above 1000 meters in the Italian Alps. Although previously utilized primarily as a local delicacy, recent studies have revealed strong antiparasitic activity through *in vitro* experiments. In Europe, numerous chemical drugs employed to combat nematodes — helminths that infest the digestive tract of livestock — are banned due to their environmental harm or show only reduced efficiency because of the development of resistance. Consequently, there is a growing demand for new alternative anthelmintic treatments in agricultural practices. Specialized metabolites found in the extracts of *C. alpina* could offer a sustainable and biological alternative to chemical drugs, specifically for nematode control. For this purpose, a unique germplasm collection originating from eight distinct natural populations in the Italian Alps was analyzed for its chemical diversity using state-of-the-art targeted LC-MS/MS spectrometry, including quantification based on multiple reaction monitoring. The predominant metabolites identified from the species were the caffeic acid derivatives chicoric acid, chlorogenic acid, and 3. 5-dicaffeoylquinic acid, the sesquiterpene lactone derivative 8-*O*-acetyl-15-*ß*-D-glucopyranosyl lactucin and the flavone glycosides, apigenin-7-*O*-glucoside and luteolin-7-*O*-glucoside, alongside their precursors apigenin and luteolin, respectively. Additionally, the genetic diversity of eighty individual plants within the germplasm collection was evaluated using ten DNA molecular markers (Simple Sequence Repeats), successfully transferred from two closely related species (*Cichorium intybus* and *Tanacetum parthenium*). This investigation unveiled a significant range of genetic diversity within the examined populations, resulting in the establishment of three distinct genetic groups. The findings were further correlated with the original ecological environment and local climate conditions spanning a biennial period, indicating substantial variations among the different accessions and the intricate interplay between genetic background and environmental factors. These results could serve as a basis for future domestication of the species through plant breeding programs ensuring product quality, but also facilitating the cultivation of *C. alpina* in more diverse geographic regions.

## 1 Introduction

*Cicerbita alpina* (L.) Wallr., syn. *Lactuca alpina* (L.) A. Gray, also known as alpine-sow-thistle, belongs to the *Asteraceae* family, specifically the subfamily *Lactucoideae* and the tribe *Lactuceae*. It is a perennial alpine herb that thrives in fertile and moist forest sites situated between 1000 and 1800 meters above sea level (Pignatti et al., 1995). With a diploid chromosome count of 2n = 18 (Doležalová et al., 2002), *C. alpina* exhibits a distinctive capitulum-type flower, like other species in the *Lactuca* and *Cichorium* genera (Pignatti, 1982). The species relies on insect pollination and primarily spreads through wind-dispersal of its seeds (Stachurska-Swakoń et al., 2012). Its distribution in Europe is sporadic, encompassing isolated habitats within central mountain ranges such as the Alpine arc, the northern Balkans, and the Carpathians (Stachurska-Swakoń et al., 2012). Notably, after the last ice age, it expanded its natural ecosystem to include Scandinavia, where it grows at lower elevations ranging from 10 to 750 meters above sea level (Michl et al., 2010). In Italy, it is distributed in the Alps and northern Apennines, and its use is prevalent in the North-Eastern regions, where locals harvest its young shoots in early spring to create unique delicacies like “Radicchio dell’Orso” and “Radic di mont” (Scartezzini et al., 2012). Until recently, its utilization was limited to such local culinary applications, food preparations, and salads. However, it has been discovered that leaf extracts from *C. alpina* exhibit potent antiparasitic activity against animal nematodes (Poulopoulou et al., 2022) which offers new application and use of the plant.

Nematodes, or roundworms, are parasitic helminths that can infect the gastrointestinal tract of livestock, leading to significant economic losses in the production chain (Roeber et al., 2013). The misuse of traditional chemical drugs employed to treat these infections has inadvertently contributed to the development of drug resistance over time, posing a challenge in modern organic agriculture, which necessitates sustainable and effective solutions for parasite control (Mendoza-De Gives, 2022). Medicinal and aromatic plants (MAPs) from ethnoveterinary-listed plants, appear to be a promising alternative to anthelmintic (AH) treatments that have been used for centuries to prevent and treat parasite infections in humans and animals by reducing the load of helminths and thereby improving the overall health of livestock. However, for many of these plants, there is a limited or incomplete scientific evaluation and validation of their efficacy, mode of action, and active metabolites (Ranasinghe et al., 2023a). In a recent study, eggs of *Ascaridia galli*, an intestinal parasite that infects poultry and is responsible for 60-84% of annual production losses (Zaman et al., 2020), were treated *in vitro* with methanolic extracts of ten different known AH plants found in the Italian Alps. Among them, *C. alpina* extract showed the lowest rates of embryonic development and was therefore selected for further studies (Poulopoulou et al., 2022).

*C. alpina* is known for its abundance of caffeic acid derivatives, including chlorogenic acid (CGA), chicoric acid (ChA), 3.5-dicaffeoylquinic acid (3.5 DCQ), and caftaric acid (Fusani and Zidorn, 2010). As a member of the *Asteraceae* family, it also accumulates sesquiterpene lactones (STLs) (Zidorn et al., 2005), which could serve as chemical/or chemosystematic markers for the tribe. STLs are bitter secondary metabolites derived from the isoprenoid/mevalonate pathway and have previously demonstrated synergistic AH activity against various nematodes (Buza et al., 2020; Shulha et al., 2020; Valente et al., 2021). Within the species, the predominant STL is 8-*O*-acetyl-15-*ß*-D-glucopyranosyl lactucin (GPL) (Fusani and Zidorn, 2010), which belongs to the guaianolide class and could show potential as an AH agent.

The present study focused on a unique collection of germplasm accessions from the province of Trentino, Italy. These accessions underwent chemical and genetic evaluation to provide new insights regarding the phytochemical profile, genetic structure, and distribution of *Cicerbita* species in northern Italy. The goal was to identify superior chemotypes and genotypes rich in potential AH substances or other antioxidant properties, which could serve as valuable starting materials for future plant breeding and domestication programs.

## 2 Materials and methods

### 2.1 Plant material and natural standards

Samples were collected from cultivated plants of *C. alpina* conserved *ex-situ* in the germplasm collection field of CREA, Research Center for Forestry and Wood located at Mount Bondone, Trento, Italy (coordinates WGS 84: E 11°02’10.8’’; N 46°01’15.7’’), at an altitude of 1534 a.s.l. in alpine environment. Eight different accessions were investigated, originating from seeds collected between the years 2004 and 2006 from as many natural populations of the species growing in the province of Trento, Italy. Details on the collection sites are reported in **Table 1**.

**Table 1:** Collection sites of seeds of investigated *C. alpina* accessions: denomination, geographic coordinates, altitude and date of collection.

N°	Denomination	Coordinates (WGS 84)		Altitude m a.s.l.	Date of collection
		N	E		
1	Agnellezza	46°11'10'	11°25'14	1501	8th August 2006
2	Bondolo	45°55'35''	10°31'05''	1840	8th September 2004
3	Juribello	46°18'10	11°45'43	1769	17th August 2005
4	Passo Manghen	46°10'27''	11°26'24''	2047	19th August 2005
5	Peller	46°19'24''	10°57'00''	1950	21th September 2004
6	San Valentino	46°04'45''	10°07'37''	1850	7th August 2006
7	Val Nambrone	46°13'00''	10°44'54''	1850	6th August 2005
8	Zambana	46°09'00''	11°01'39''	1777	26th August 2004

In June 2015, the plantlets were transplanted into the field, arranged in single rows with row-to-row and within-row spacing of 0.8 meters and 0.4 meters, respectively. Subsequently, the plants were cultivated following conventional agricultural practices, encompassing mechanical weed control between rows and manual weed removal within rows, periodic irrigation as required, and the complete avoidance of fertilizers and pesticides.

At the end of May 2021, individual plants per accession (eighty in total) were randomly chosen and labeled with metal sticks for successive sampling for genotyping and chemical analysis: Agnellezza (10 representative plants), Bondolo (13), Juribello (9), Passo Manghen (9), Peller (13), San Valentino (9), Val Nambrone (9), and Zambana (11). On May 27th, 2021, fresh leaf material from each labeled individual was collected and directly frozen in liquid nitrogen for subsequent DNA extraction. Later, in July 2021 and July 2022, the whole aerial parts of the same selected plants (leaves, stems and flower heads) were harvested at full blooming stage and dried with the aim of characterizing their metabolite profile (for details see 2.5). Samples were dried in a thermostatic oven at 38 ± 1°C for 48 h at CREA laboratories and successively placed in paper bags until further use. Collected samples were authenticated by P. Fusani of CREA of Trento, Italy, where voucher specimens of the species were deposited (CA#202201-02).

Analytical standards were obtained from Phytolab (Vestenbergsgreuth, Germany) and TransMIT PlantMetaChem (Giessen, Germany). GPL was a kind gift from Prof. Dr. Christian Zidorn (Kiel, Germany).

### 2.2 DNA extraction and SSR marker optimization

For the genomic DNA extraction, 30 mg of lyophilized plant material (*C. alpina* leaves) was weighed and processed following the DNeasy 96 Plant Kit protocol by Qiagen (Hilden, Germany). The DNA concentration was determined using a NanoDrop ND-8000 spectrophotometer (NanoDrop Technologies, Thermo Scientific, Wilmington, DE, USA) at a wavelength of 260 nm. To assess genetic diversity, *C. intybus* SSR markers developed at 24 loci by Patella et al. (2019) and *Chrysanthemum nankingense (Asteraceae)* SSR markers developed at 10 loci by Wang et al. (2013), which had been successfully transferred to *T. vulgare* (unpublished data, Martinidou), were applied to the DNA samples of *C. alpina* (**Supplementary Table S1**).

PCR amplifications were performed following the tailing PCR method (Schuelke, 2000), which includes three different primers: a pair of locus-specific primers (**Suppl. Tab. S1**) one of which had an oligonucleotide tail at the 5’ end, and a third primer (from now on called the Tail) complementary to the Tail and labeled with a fluorescent dye (e.g., HEX, TAM, ROX, FAM, respectively). The PCR was carried out in a 20 μl reaction volume with PCRBIO HS Taq Mix (3 mM MgCl2, 2 mM dNTPs, enhancers, and stabilizers), 0.25 M of the non-tailed primer, 0.75 M of the tailed primer, 0.5 M of the tail, and 10 ng of template DNA. PCR amplifications were performed in a BIO-RAD thermal cycler (PCR Biosystems Inc., Wayne, Pennsylvania, USA) with the following conditions: for group 1a, the initial denaturation was set at 94°C for 5 min, followed by a touchdown for 3 cycles at 94°C for 30 s, an annealing step at 56°C for 30 s, decreasing by 1°C per cycle (56-54°C), and an extension at 72°C for 30 seconds. The next 35 cycles were performed at 94°C for 30 s, at 54°C for 30s, and at 72°C for 30 s, with a final extension at 72°C for 15 min. For group 1b: 94°C for 5 min, followed by a touchdown for 8 cycles of 94°C for 30 s, an annealing step at 61°C for 30 s decreasing by 1°C per cycle (from 61 to 54°C), and an extension at 72°C for 30 s. The annealing temperature for the following 37 cycles was set to 54°C, with denaturation and extension phases as above and a final extension at 60°C for 30 min.

For the three loci derived from *T. parthenium*, PCR was conducted using the same reaction volumes, but the amplifications were carried out under different thermal cycling conditions. In all groups, the process began with an initial denaturation step at 94°C for 3 min, followed by ten cycles of denaturation at 94°C for 30 s. For group 2a, the annealing temperature ranged from 57to 62°C for 30 s, for group 2b it ranged from 52 to 57°C, and for group 2c it ranged from 50 to 55°C, with an incremental increase of 0.5°C in the annealing temperature for each cycle. A final extension step was performed at 72°C for 30 s. The subsequent 28 cycles consisted of denaturation at 94°C for 30 s, annealing at the specific temperature for each group for 30 s, and extension at 72°C for 30 s. The amplification process had a final elongation step of 72°C for 7 min.

In the next steps, the PCR products were identified on a 1.2% agarose gel and the reproducibility of the PCR methods was tested multiple times before finalizing the procedure. The PCR reaction was purified using sodium acetate–ethanol DNA precipitation. One microliter of the purified sample was analyzed on a capillary electrophoresis system, Agilent 2100 Bioanalyzer, with the DNA7500 Kit (Agilent Technologies, Santa Clara, CA, USA) according to the manufacturer’s instructions. The allele’s size was verified through GeneMarker 3.0 (Holland and Parson, 2011). The loci that produced clear polymorphic bands were used in the genetic analysis of *C. alpina*.

### 2.3 Genetic data analysis

A data matrix was created using the different allele sizes at different loci and analyzed with GenAlex version 6.1 software (Peakall and Smouse et al., 2012) to estimate genetic diversity by the use of parameters such as the number of different bands, the number of different bands with a frequency higher or equal to 5%, the number of private bands, the number of common bands found in 25% or fewer populations, the number of common bands in 50%, and the mean of expected heterozygosity (He) for each population. Moreover, the software was used to generate a pairwise individual-by-individual (NxN) genetic distance matrix and calculate the variance components (degrees of freedom (df), Sum of Squares (SS), Mean Sum of Squares (MS), estimated variance, and conversion of estimated variances to percentage of total variance). The number of permutations for significance testing was set at 9999. Finally, the genetic distance matrix was used to perform subsequent PCA analyses.

The software STRUCTURE 2.3.3 (Pritchard et al., 2000; Falush et al., 2003), which, through iterative algorithms, identifies clusters of related individuals from multilocus genotypes, was applied to examine the genetic structure of populations. Ten independent runs of STRUCTURE were performed for each K value from 1 to 10. Each run consisted of a burn-in period of 100.000 steps, followed by 1.000.000 Monte Carlo Markov Chain replicates, assuming an admixture model and correlated allele frequencies. No prior information was used to define the clusters. The most likely K was chosen, using STRUCTURE Harvester, comparing the average estimates of the likelihood of the data, ln (Pr(X/K)), for each value of K (Pritchard et al., 2000), as well as calculating the *ad hoc* statistics ΔK, based on the rate of charge in the ln-probability of data between successive K values (Evanno et al., 2005).

### 2.4 Canonical correspondence and principal component analysis

Canonical correspondence analysis (CCA), Principal Components Analysis/Hierarchical Clustering Analyses (PCoA) were performed using Past software v. 2.17c (Hammer et al., 2001). PCoA can help identify relatively homogeneous groups within the germplasm collection of a plant species based on the genetic results obtained, while CCA is a multivariate method to elucidate the relationships between biological assemblages of species and their environment. This analysis correlates species composition with different predictive variables (Ter Braak, 1986), and in the present study it has been used to describe the relationship between environmental variables and genetic composition (Angers et al., 1999; Dell'acqua et al., 2014).

The analysis was performed, using an environmental variables/genetic data matrix, on the mean data for different compound classes and genotyping data of each population, aiming to investigate the presence of clusters because of differences in environmental conditions (temperature, rain, and altitude) and or the genotype. The ordination axes are linear combinations of the environmental variables. The first three input file columns contain two years (2021 and 2022) of environmental variables/data. Each population has been assigned a different color and acronym (Agnellezza: black/A_2021-A_2022; Bondolo: aqua/B_2021-B_2022; Juribello: blue/J_2021-J2022; Manghen: blueviolet/M_2021-M_2022; Val Nambrone: chartreuse/N_2021-N_2022; Peller: orangered/P_2021-P_2022; San Valentino: cadetblue/V_2021-V2022; Zambana: forestgreen/Z_2021-Z_2022).

### 2.5 Chemical extraction and LC-MS/MS method establishment for sesquiterpene lactone and caffeic acid derivatives

The aerial parts of the plants were dried at 36°C (+/-1°C) for 60 hours, followed by a day at 38°C (+/-1°C). Each sample was ground separately into powder for chemical characterization using an IKA grinder (IKA® A11, Sigma Aldrich) [Bioanalytical Method Validation Guidance for Industry (2018)]. Approximately 100 milligrams of thoroughly blended and desiccated plant material were accurately measured and placed into 15 ml tubes. To create the extracts, 4 ml of a mixture consisting of 80% methanol (MeOH) in H_2_O was added. The solvent also included a concentration of 2 parts per million (ppm) of *trans*-cinnamic acid D5, serving as the internal standard. The samples were sonicated at room temperature for 20 min and rotated for 1 hour before filtration with MILLEX 13-0.22 µm PTFE filters (Merck-Millipore, Darmstadt, Germany).

To establish a precise targeted methodology for the chemical analysis of metabolites in *C. alpina*, pure analytical standards at a concentration of 5 ppm as listed in **Supplementary Table 2** were used. These standards were infused to fine-tune the Mass parameters, specifically focusing on the identification of the parent compound (Q1) as well as its corresponding products (Q3). The following values were documented: the DP (declustering potential), the EP (entrance potential) of the product ion, along with the CE (collision energy/the voltage that produces each fragment), and the CXP (cell exit potential) for each ion product. Finally, an acquisition method was set up, and a mix of all metabolites was injected into the UHPLC to record the RT (retention time) of each component (**Supplementary Table S2**).

The UHPLC system was coupled directly to an API 5500 Triple Quadrupole Mass spectrometer (Applied Biosystems/MDS Sciex, Toronto, Canada) equipped with an electrospray source. Analyst™ software version 1.6.1 was used for instrument control and data acquisition. The transitions and spectrometric parameters were optimized individually for each standard compound by direct infusion of their solutions (5 ppm, MeOH/H2O (80:20 v/v)) into the mass spectrometer at a flow rate of 20 μL per min.

### 2.6 Instrumental conditions

Separation was performed using a UHPLC Dionex 3000 (Thermo Fisher Scientific, Germany), with an Acquity UPLC BEH C18 1.7 μm column 2.1 x 150 mm purchased from Waters (Milford, MA, USA) with a relative pre-column. The column temperature was set at 45°C. Samples (2 μl) were injected using an autosampler set at 10°C. The flow rate was 0.350 ml/min, and the composition of the mobile phase was: solvent A - water with 0.5% formic acid, and solvent B - acetonitrile with 0.5% formic acid. Separation was carried out following an eighteen-minute gradient. Specifically, during the first 10 min, the concentration of solvent B was gradually increased from 5% to 50%, followed by a rapid increase to 100% over 2 min. The mobile phase composition remained at 100% B for 2 min before being decreased to 5% in 1 min. Finally, the column was re-equilibrated at this level for an additional 3 min.

The spray voltage was set at 5500 V for positive mode and -4500 V for negative mode. The source temperature was set at 400°C, and the nebulizer gas (Gas 1) and heater gas (Gas 2) were at 40 and 30 psi, respectively. UHP nitrogen (99.999%) was used as both curtain and collision gas (CAD) at 20 and 9 psi, respectively.

### 2.7 Method validation

The validation of the method was performed following the accepted US Food and Drug Administration (FDA) bio-analytical method validation guide (US Department of Health and Human Services) (2018). The assays were established on calibration standards and quality control samples extracted from *C. alpina* with the provided protocol (80% MeOH in H_2_0), to test variances like linearity, the limit of detection and quantification, the matrix effect, the intra and inter-day variability and finally the recovery rate of the method.

Calibration curves were prepared in pure solvent (80% MeOH), and in matrix extracts. To assess the percentage of matrix effect for each of the fifteen studied substances, the calibration curves were compared. The limits of quantification (LoQ) and linearity range were determined at concentrations where the quantifier transition had a signal-to-noise ratio greater than 10 (**Supplementary Tables S3A, B**).

To determine the intra-day and inter-day variability, ten replicate quality control samples of *C. alpina* were extracted, injected on the same day, and then re-injected for seven consecutive days. Intra-day and inter-day variability were evaluated by calculating the coefficients of variation (CV %) as shown in **Supplementary Table S3B**.

The quality control samples were further used for method validation by spiking or supplementing them with known amounts (1 ppm, 5 ppm) of each substance. The recovery test was estimated on ten spiked *C. alpina* samples and calculated as the average of the “measured value/expected value” ratio (%) (**Supplementary Table S3B**). The applicability of the proposed analytical method was tested in 454 samples of *C. alpina* for the identification of their STLs and caffeic acid derivatives content.

### 2.8 Polyphenolic content

Targeted Ultra High-Performance Liquid Chromatography (UHPLC) was performed on a Waters Acquity System (Milford, MA, USA) having a binary pump, an online vacuum degasser, an autosampler and a column compartment with the same extracts as analyzed for STLs and caffeic acid derivatives (see 2.5). Separation of the compounds was achieved on a Waters Acquity HSS T3 column 1.8 μm, 100 mm × 2.1 mm, at 40°C. The analysis of phenolic compounds was performed according to Vrhovsek et al. (2012). For Mass Spectrometry (MS) detection, a Waters Xevo TQ MS instrument equipped with an ElectroSpray (ESI) source was used.

### 2.9 Data processing and statistical analysis

Data processing was performed using the MassLynx and Target Lynx Application Manager (Waters) and MultiQuant. Variations in quantified compounds between accessions were evaluated using ANOVA (One Way and Two Way Anova, check for normal distribution), followed by the Student–Newman–Keuls test for multiple comparisons to determine if p was smaller than 0.05. Statistical analyses were performed using Statistica for Windows software and Jamovi. Means, standard deviations, and correlations were calculated using MS Excel 2016.

## 3 Results

### 3.1 Genetic results

#### 3.1.1 Genetic variability evaluation

**Figure 1:**
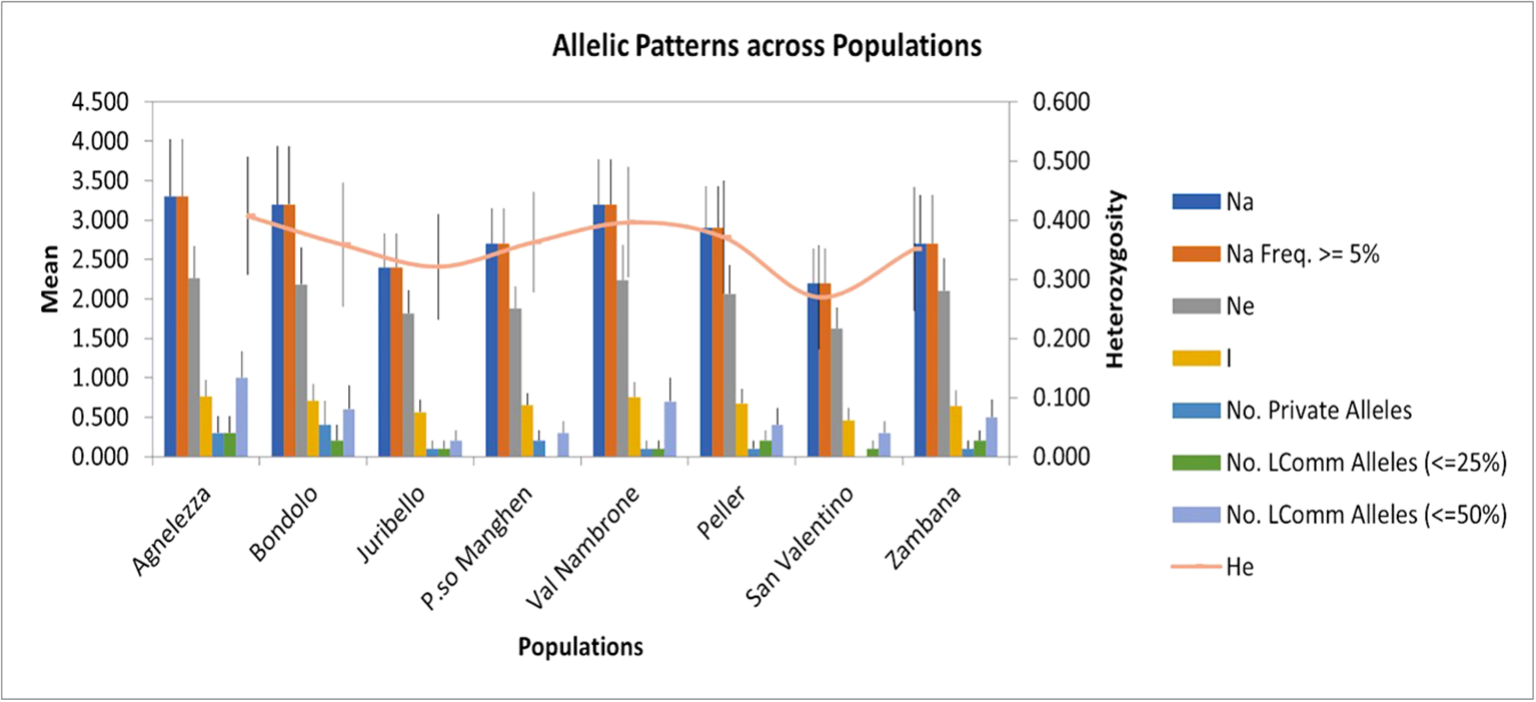
The figure was generated using the data presented in **Table 2** (mean allelic patterns across populations). The SSR study was carried out to investigate the genetic parameters for the germplasm collection of *C. alpina.* The highest mean of expected heterozygosity (He) was observed for Agnelezza (0.41), while the lowest was for San Valentino (0.26).

To evaluate the genetic structure of the accessions, SSR markers of closely related species were tested for their transferability. Out of 24 primers developed for *C. intybus*, five gave no amplification products and another twelve were unspecific; therefore, they were not further considered in the genetic evaluation. Finally, seven SSRs (M1.2, M7.19, M5.14, M4.11b, M4.10a, M2.6, and M1.3) (Patella et al., 2019) could be successfully applied to *C. alpina*. Furthermore, three loci (89, 135, and 341) out of ten tested could be transferred from *T. parthenium* (Wang et al., 2013). In the end ten specific markers were successfully optimized and used for the characterization of genetic diversity in eight populations of *C. alpina* (**Supplement Table S1**). In **Figure 1**, the variations in the amplified allelic patterns across the germplasm collection are presented (for raw data see **Table 2**). In total, 53 different alleles were detected for the eight accessions. The average number of different alleles (Na) ranged from 2.00 to 3.30, with an average of 2.5 alleles per locus examined. The highest mean of expected heterozygosity (He) within the different accessions was observed for Agnelezza (0.41), a collection originating from lower altitudes within the Lagorai mountain chain. In contrast, the lowest was observed for San Valentino (0.26), an ‘isolated’ collection of the Adamello region (see **Figure 1**). In general, the unbiased heterozygosity values were higher than the numbers of He for all the populations, probably due to the small population sizes (Harris and DeGiorgio, 2017). The Shannon-Weaver information index (I) ranged from 0.45 in Passo Manghen and San Valentino to 0.77 in Agnelezza, the collection’s highest genetic variability. The most polymorphic locus for *C. alpina* was M4.10a, while unique alleles (Private Alleles) were detected for the accessions Agnelezza, Bondolo, Juribello, Val Nambrone, Peller, and Zambana and could be used in future breeding programs, for the segregation of those accessions.

**Table 2:** The mean allelic pattern found for each of the eight *C. alpina* populations during the SSR tests are depicted below.

Population	Agnelezza	Bondolo	Zuribello	Passo Manghen	Val Nambrone	Peller	San Valentino	Zambana
Na	3.300	3.400	2.300	2.000	2.600	3.300	2.100	2.700
Na Freq. >= 5%	3.300	2.700	2.300	2.000	2.600	2.600	2.100	2.600
Ne	2.261	2.138	1.830	1.671	1.875	2.180	1.616	2.058
I	0.766	0.753	0.556	0.452	0.623	0.723	0.448	0.634
No. Private Alleles	0.300	0.700	0.200	0.000	0.100	0.100	0.000	0.100
No. LComm Alleles (<=25%)	0.500	0.200	0.000	0.000	0.000	0.300	0.100	0.200
No. LComm Alleles (<=50%)	1.000	0.700	0.300	0.300	0.600	0.900	0.400	0.700
He	0.407	0.392	0.323	0.270	0.353	0.375	0.264	0.348
uHe	0.430	0.409	0.349	0.304	0.377	0.391	0.284	0.369

The reported information are: Na = Number of Different Alleles, Na (Freq >= 5%) = No. of Different Alleles with a Frequency >= 5%, Ne = No. of Effective Alleles = 1 / (Sum pi^2), I = Shannon's Information Index = -1* Sum (pi * Ln (pi)), No. Private Alleles = No. of Alleles Unique to a Single Population, No. LComm Alleles (<=25%) = No. of Locally Common Alleles (Freq. >= 5%) Found in 25% or Fewer Populations, No. LComm Alleles (<=50%) = No. of Locally Common Alleles (Freq. >= 5%) Found in 50% or Fewer Populations, He = Expected Heterozygosity = 1 - Sum pi^2, uHe = Unbiased Expected Heterozygosity = (2N / (2N-1)) * He, pi= number of affected Alleles.

#### 3.1.2 Population structure analysis of *C. alpina*

**Figure 2:**
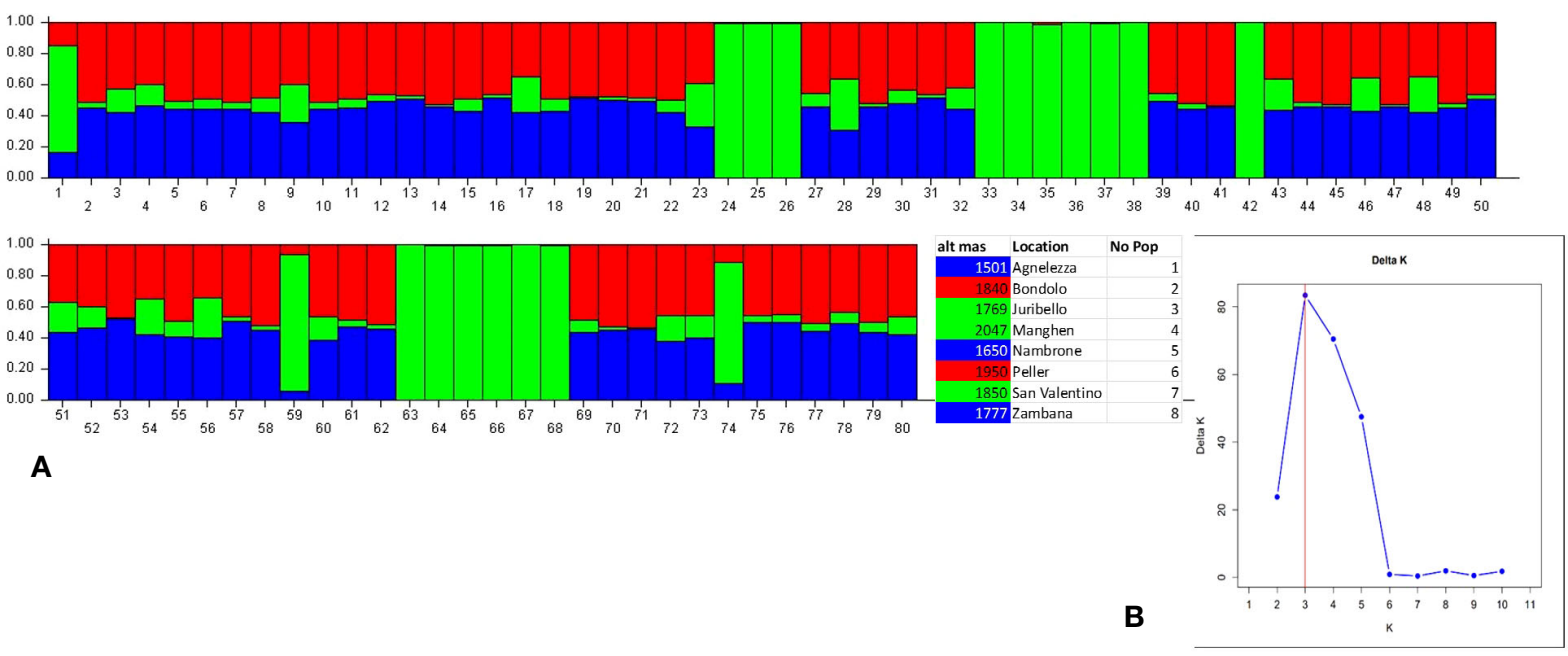
Bayesian model-based structure (K=3) **(2B)** of 80 individual plants of *C alpina* originating from eight germplasm collections distributed in the Italian Alps. The barplot represents each individual as a single vertical bar broken into K color segments **(A)**, with lengths proportional to the estimated probability of membership in each inferred cluster (Cluster A: Blue, Cluster B: Green, Cluster C: Red).

**Figure 3:**
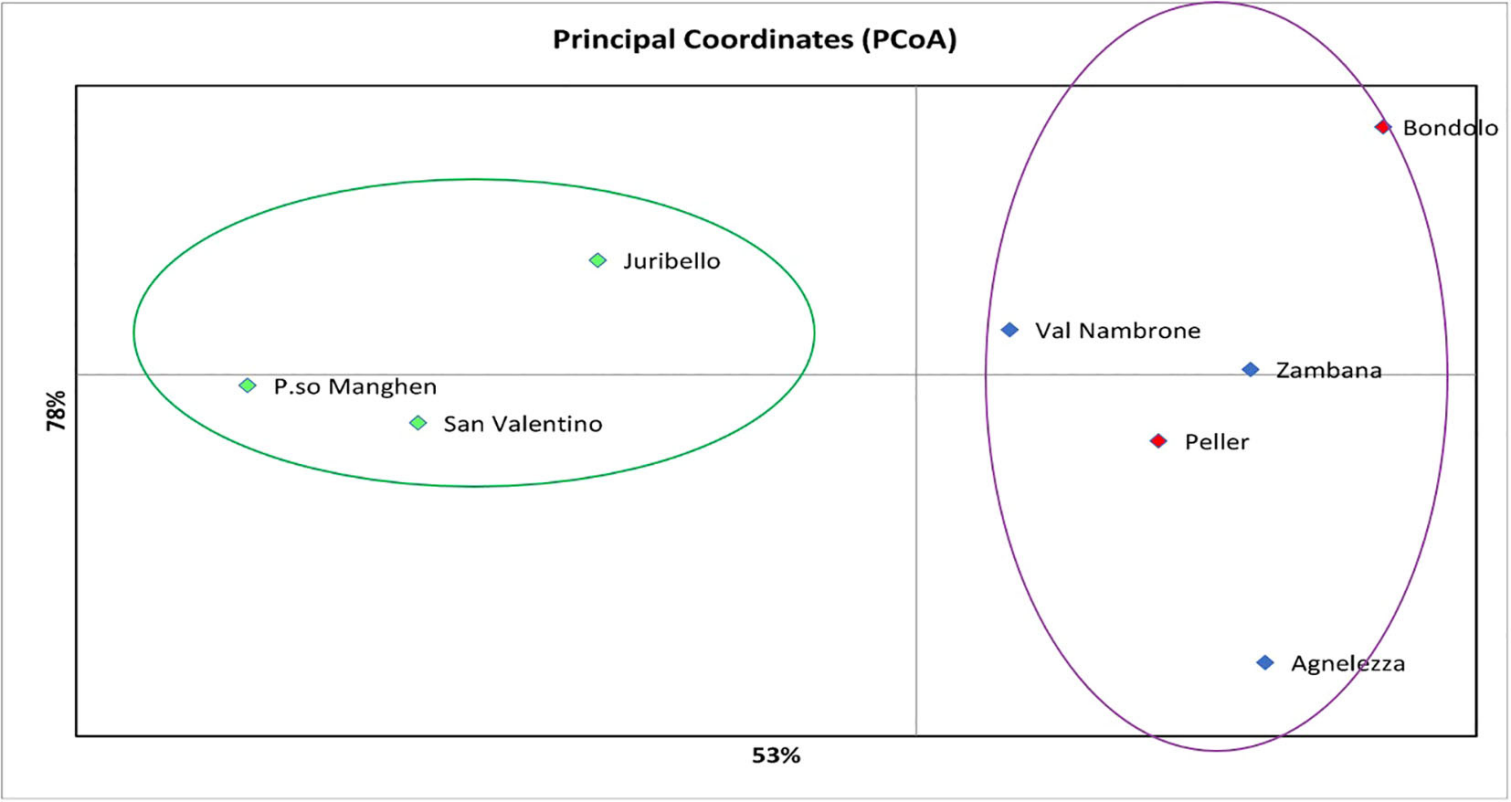
The Hierarchical Clustering analysis (PCoA) presents/recognizes two different clusters between the eight local populations of *C. alpina.* The first one would be the green cluster consisting of accessions that belong to the genetic pool B (Passo Manghen, Juribello, and San Valentino), while the second one concentrates the remaining individuals that are an admix of the genetic pools A and C (Bondolo, Val Nambrone, Peller, Zambana and Agnelezza).

Structure analysis assigns genotypes into respective groups according to their allele frequencies. In **Figure 2A**, each analyzed individual is represented by a single vertical line, and each color represents a different gene pool as calculated by STRUCTURE, presuming that each individual has inherited a distinct proportion of its ancestor from each of the K genotypes (Pritchard et al., 2000). In the case of the eight *C. alpina* germplasms, the STRUCTURE analysis assumed three genetic groups (K = 3) as the most likely number (**Figure 2B**). Considering the mean value for each geographical group, a PCoA was constructed with the help of GenAlex v. 6.1 and PAST v. 4.03 software. The Hierarchical Clustering analysis (PCoA) was done to assess the presence of possible clusters between and within the eight different populations. Through this process, two different clusters were determined: Juribello, Paso Manghen, and San Valentino assigned to the genetic group B (green) formed a separate class of their own, while the remaining accessions formed a second class (genetic groups A and C) (**Figure 3**).

**Figure 4:**
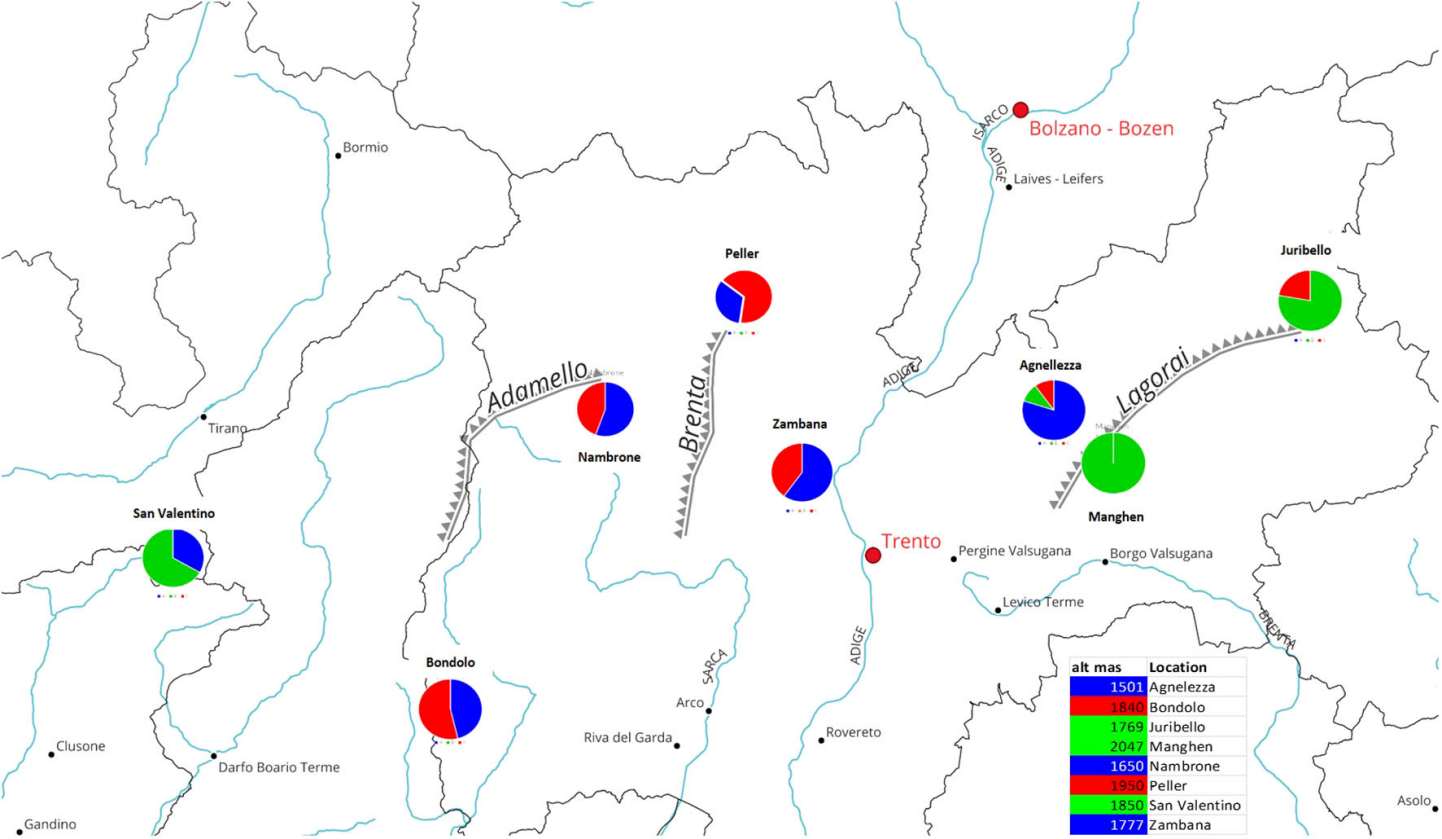
The circles show the percentage of ancestry (for K=3) and the geographic origin within the province of Trentino (Italy) of the eight different *C. alpina* accessions. More specifically, two of the ancestors (the blue and red clusters) depicted admixed individuals with a variable percentage of each membership in them (blue, green, and red), while only individuals of the second green cluster exhibited pure representatives. It is noteworthy that 2 of the admixed accessions (Peller and Bondolo) showed a higher percentage of the red cluster, while Val Nambrone, Zambana, and Agnelezza exhibit a higher percentage of the blue cluster. Except for Passo Manghen, which was 100% derived from Cluster B (green), San Valentino and Juribello (both predominantly green) presented lower percentages of the blue and red clusters, retrospectively.

The genetic analysis confirmed that accessions originating from the center of the region, eastern or western of the Adige River (Peller, Val Nambrone, Bondolo, Zambana, and Agnellezza) are a genetic mixture between group A and C (red and blue), as can be seen in the geographic map (**Figure 4**). Contrariwise, Passo Manghen, Juribello, and San Valentino originate from germplasm collections found in the middle and eastern parts of the Lagorai mountain and in Adamello, located on the western border between Trentino and Lombardia, respectively, and they predominantly belong to the genetic cluster B (green). A link between genetic structure and the origin could be proposed in relation to the Adige River and the populations distributed within the mountainous chain of Adamello and the southern parts of Lagorai. Finally, there might be a correlation between the genetic groups and the geographic parameter of altitude: since populations like Agnellezza, Val Nambrone, and Zambana, which were found to belong principally to the first genetic group (blue) were sampled from lower altitudes than the rest of the populations (**Figure 4**); however, a more broaden genetic analyses would be helpful to further support that assumption.

### 3.2 Chemical results

#### 3.2.1 Development and validation of a new LC-MS/MS-based method for qualitative and quantitative analysis of STLs and caffeic acid derivatives

The optimization of the instrument parameters was achieved by infusion of the standard compounds in the Mass Spectrometer and in accordance with the manufacturer’s recommendations. Two classes of compounds could be identified in the method: (1) STLs and (2) caffeic acid derivatives. The relevant transitions and parameters were manually optimized as shown in **Supplementary Table S2**. The fragmentation pattern of each metabolite was studied by comparing the results with the existing literature if available. Four DCQ derivatives (1.3 DCQ, 3.4 DCQ, 3.5 DCQ, 4.5 DCQ), caftaric acid, CGA and ChA, the STLs lactucin (LAC) and lactucopicrin (LACP), and the carboxylic acids (tartaric and shikimic acid) were identified in negative mode using [M–H] − as precursor ion, while the LAC derivative GPL was found in the [M+HCOO] − adduct ion. In contrast, *p*-coumaric and caffeic acid plus costunolide were characterized by a positive ionization mode [M+H] +.

The method validation process adhered to the established guidelines outlined in the US Food and Drug Administration (FDA). Calibration standards and control samples derived from *C. alpina* were utilized to evaluate crucial validation factors (as shown in **Supplementary Table S3**). To assess the linearity of the instrument’s response calibration curves of the fifteen studied compounds were employed over a broad concentration range (0.0002 ppm to 100 ppm). The outcomes, encompassing the ranges of linearity, curve parameters, and limits of detection (LODs), can be found in **Supplementary Table S3A**. Notably, 1.3 DCQ, GCA, and costunolide exhibited the lower LODs (0.0002 ppm) in the study, while most compounds had a range that did not exceed 10 ppm. Additionally, the matrix effect [1-(SlopSolvent/SlopMatrix)*100] was accounted for by comparing each analyte’s response in the sample matrix to its response in a standard solution and expressed as a percentage. All the studied compounds displayed positive values indicating ion suppression, except costunolide (S3a) that have shown ionization enhancement. For compounds, where the matrix effect exceeded the suggested value (15%), the repeatability and recovery test were acceptable (<15%, as can be seen in **Supplementary Table S3B**) and for this reason, they were not excluded from the final method.

To evaluate variability within a single day (intra-day) and across consecutive days (inter-day), ten replicate quality control samples of the plant were extracted and injected on the same day, followed by reinjection for seven consecutive days. According to existing literature, the coefficient of variation (CV %) should not exceed 15% for intra-day assays and 20% for inter-day assays. As observed in **Supplementary Table S3B**, only CGA approached the recommended limit of 15% (Reed et al., 2002). Lastly, a recovery test was conducted by spiking ten samples with known amounts of the studied substances (1 ppm and 5 ppm). Recovery was determined by calculating the percentage of the average ratio between the measured value and the expected value. The results are presented in **Supplementary Table S3B** and are higher than 90%.

#### 3.2.2 STLs and caffeic acid derivatives

To gain a better understanding of the chemical diversity of *C. alpina*, a thorough chemical analysis was conducted using the novel developed and validated targeted metabolite profiling approach based on LC-MS/MS including MRM-based quantification. To evaluate the phytochemical profile and the bioactive potential of *C. alpina*, the main classes of compounds described for the species (i.e., STLs and caffeic acid derivatives) were analyzed qualitatively and quantitatively.

**Figure 5:**
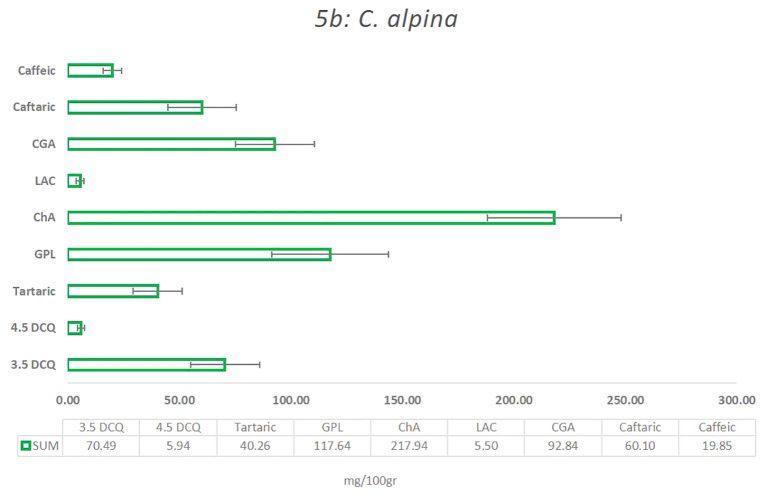
The mean value of the most significant caffeic acid and STLs derivatives that were identified in the *C alpina*, and how they differentiate among the eight Populations for the years 2021 and 2022 (A, Agnelezza; B, Bondolo; J, Juribello; M, Manghen; N, Val Nambrone; P, Peller; V, San Valentino; Z, Zambana) alongside their calculated standard deviation (SD) error.

**Figure 6:**
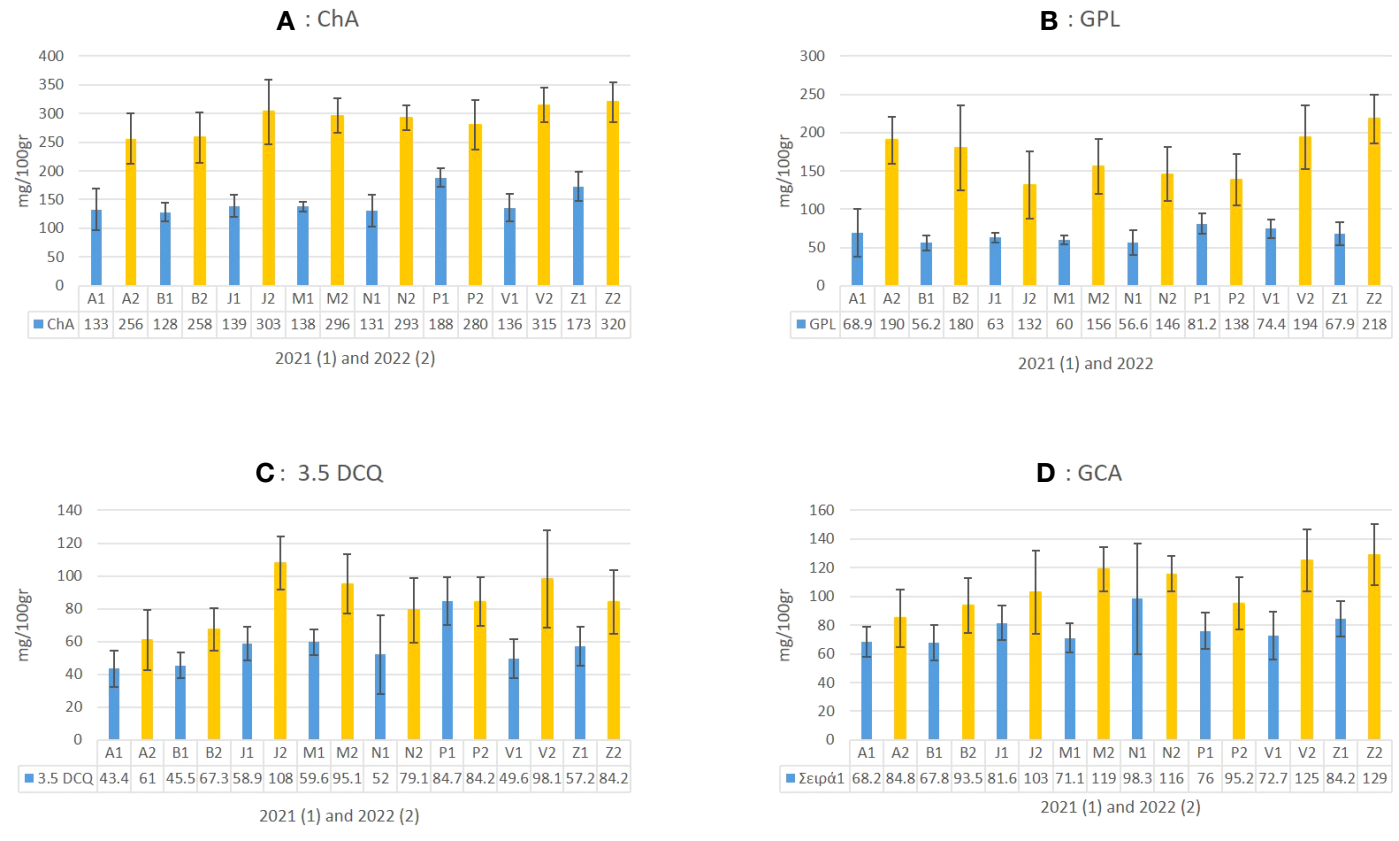
The mean value of four abundant metabolites in *C. alpina* (**A**: ChA, **B**: GPL, **C**: 3.5 DCQ, and **D**: CGA) and how they differentiate between the eight populations for the years 2021 (1) and 2022 (2) are presented in the above figure.

In the present study, the main compounds identified in *C. alpina* were: 4.5 DCQ, 3.5 DCQ, ChA, GPL, caftaric acid, caffeic acid, LAC, and traces of LACP, and 3.4 DCQ, as well as the two carboxylic acids shikimic and tartaric acid, respectively, as shown in **Figure 5** and **Supplementary Table S4A**. Among them, the metabolites with the highest concentration were ChA, followed by GPL, CGA, 3.5 DCQ, caftaric, tartaric, and finally caffeic acid (**Figure 6**).

Several studies support the assumption that certain STLs themselves or in synergy with other natural substances are responsible for the AH activity of plants and plant extracts (Valente et al., 2021; Ranasinghe et al., 2023b). For this reason, special attention was given not only to the main STL derivative (GPL), but also to LAC, LACP, and other known insecticidal metabolites, such as ChA, CGA, and 3.5 DCQ during the chemical analyses of *C. alpina* (Valente et al., 2021; Ocampos et al., 2023). According to the analysis, the eight accessions were all rich in ChA, GPL, CGA, 3.5 DCQ, and caftaric acid. The mean values of the four most prevalent substances and how they differentiate between the eight populations over the years 2021 and 2022 are depicted in **Figure 6**. The most dominant substance was found to be ChA, followed by the STL derivative GPL and CGA, which is partially in agreement with the findings of Fusani and Zidorn (2010).

Chemical analyses of plant material in 2021 and 2022 showed a significant increase for most of the analyzed metabolites. For example, ChA levels almost doubled from 172.5 mg/100 g of dry plant material (mean value of 2021) to 315 mg/100 g (mean 2022) in the second year. A similar trend was observed for GPL, 3.5 DCQ, and other compounds (**Figure 6**). Concerning the accumulation of ChA during the second year (2022), the populations of Zambana and San Valentino produced the highest amounts (320 and 315 mg/100 g), followed by Juribello, Passo Manghen, Val Nambrone, and Peller, for which no significant statistical difference could be found. In 2021, Peller and Zambana accumulated the highest amounts of ChA, followed by Juribello, Passo Manghen, San Valentino, Val Nambrone, Bondolo, and finally Agnelezza. Bondolo and Agnellezza had lower concentration levels during both years (**Figure 6A**).

Regarding the main STL derivative, GPL, populations Peller and San Valentino accumulated the highest values in 2021, followed by Zambana and Agnelezza, while in 2022 Zambana produced the highest amount (218 mg/100gr). Furthermore, San Valentino, Agnelezza, and Bondolo showed an accumulation level between 194 and 180 mg/100 g of tissue, while the remaining population showed significantly lower amounts (132–156 mg/100 g) (**Figure 6B**). The accumulation of the most abundant DCQ isomer in *C. alpina*, 3.5 DCQ, was found to be highest in 2022 in accessions Juribello, San Valentino, Val Nambrone, and Zambana (**Figure 6B**). In contrast with the general trend observed in the collection, Peller was the only accession that presented similar values of the substance for both years and was the higher producer of 3.5 DCQ during 2021 (84.7 mg/100 g). Zambana and San Valentino presented the highest amounts of CGA in 2022, while during the first year, it was Val Nambrone.

To summarize, Zambana, San Valentino, and Juribello produced the highest amount for most of the studied substances during the second year of evaluation, while during the first year, it was Peller that seemed to differ among the other accessions. A two-way ANOVA confirmed the significant difference (p ≤ 0.05) between each dependent variant (metabolite) and our fixed factors (time, population, and time* population), as can be given for GPL in **Supplementary Figure S1**. As a general trend from the chemical analysis, it could be stated that each substance was found to increase substantially during the second year of evaluation (2022). For some metabolites, such as LAC and caffeic acid (S1a and S1b), the concentration between 2021 (blue) and 2022 (yellow) changed significantly, with an observed ten-fold (LAC, Juribello) or even forty-fold (caffeic acid, Zambana) increase (**Supplement Figure S1A&B**). Both are intermediate to either GPL or caffeic acid derivatives, respectively. Their temporary accumulation indicates some disturbance in the biosynthesis and the related metabolite flux. Furthermore, since GPL derives from LAC (Liu, 2013) it is not a surprise that an increase in its direct precursor would also have a positive effect on the final amount of the product. Similar situations can be seen for caffeic acid and its derivatives. For example, the concentration of ChA, and CGA was significantly higher in 2022 and correlated to some extent with the concentration of their precursors.

#### 3.2.3 Polyphenolic content

**Figure 7:**
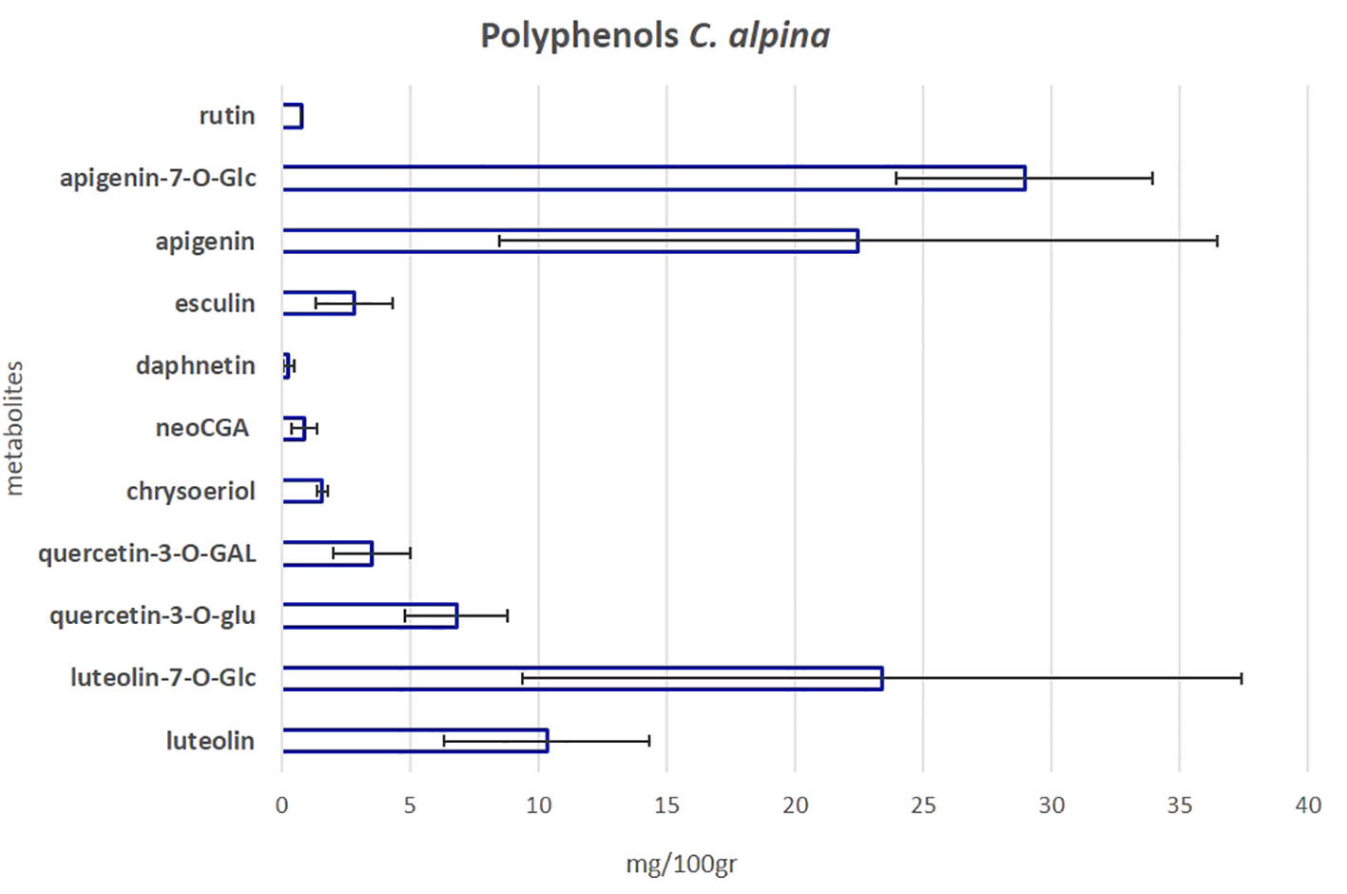
The mean value of the eleven most significant polyphenols in *C. alpina* along with the calculated SD error bars. The value was calculated as the mean accumulated for each metabolite in the years 2021 and 2022.

To investigate the polyphenolic profile of the *C. alpina* accessions, the samples were further subjected to a previously established targeted LC-MS/MS method (Vrhovsek et al., 2012). In total, eleven polyphenols were detected and quantified. The main additional phenolic compounds isolated were the flavone glycosides, apigenin-7-*O*-glucoside (Ap-7-O-glu) and Lu-7-*O*-glucoside (luteolin-7-O-glu), alongside their precursors apigenin (Ap) and luteolin (Lu), the methylated flavone chrysoeriol, the flavonol glycosides quercetin-3-*O*-glucuronide (quercetin-3-*O*-glu), quercetin-3-*O*-galactoside (quercetin-3-*O*-GAL), rutin (syn. quercetin 3-*O*-rutinoside) and the CGA acid derivative neochlorogenic acid (neoGCA). Furthermore, two coumarins, esculin, and daphnetin (only in traces), plus ferulic acid (only in traces) were identified. Among them, the most highly accumulated metabolites were Ap-7-*O*-gluc, Lu-7-*O*-glucoside, Ap, and Lu, as can be seen in **Figure 7** and **Supplementary Material Table S4B**. Similar results have been recently published by the group of Zheleva-Dimitrova et al. (2023). According to Ocampos et al. (2023) the AH activity of *A. absinthium*, a medicinal plant of the *Asteraceae* family with deworming and insecticidal properties (Szopa et al., 2020), could be mainly attributed to its polyphenolic compounds, without dismissing the fact that other metabolites may also be present and play a crucial role. Among them, apigenin was one of the identified active flavones. Ap is possible to inhibit larva growth as has been shown before for *Caenorhabditis elegans*, a nematode commonly used for mode-action studies of anthelmintics and nematicides (Yoon et al., 2006; Holden-Dye and Walker, 2014).

**Figure 8:**
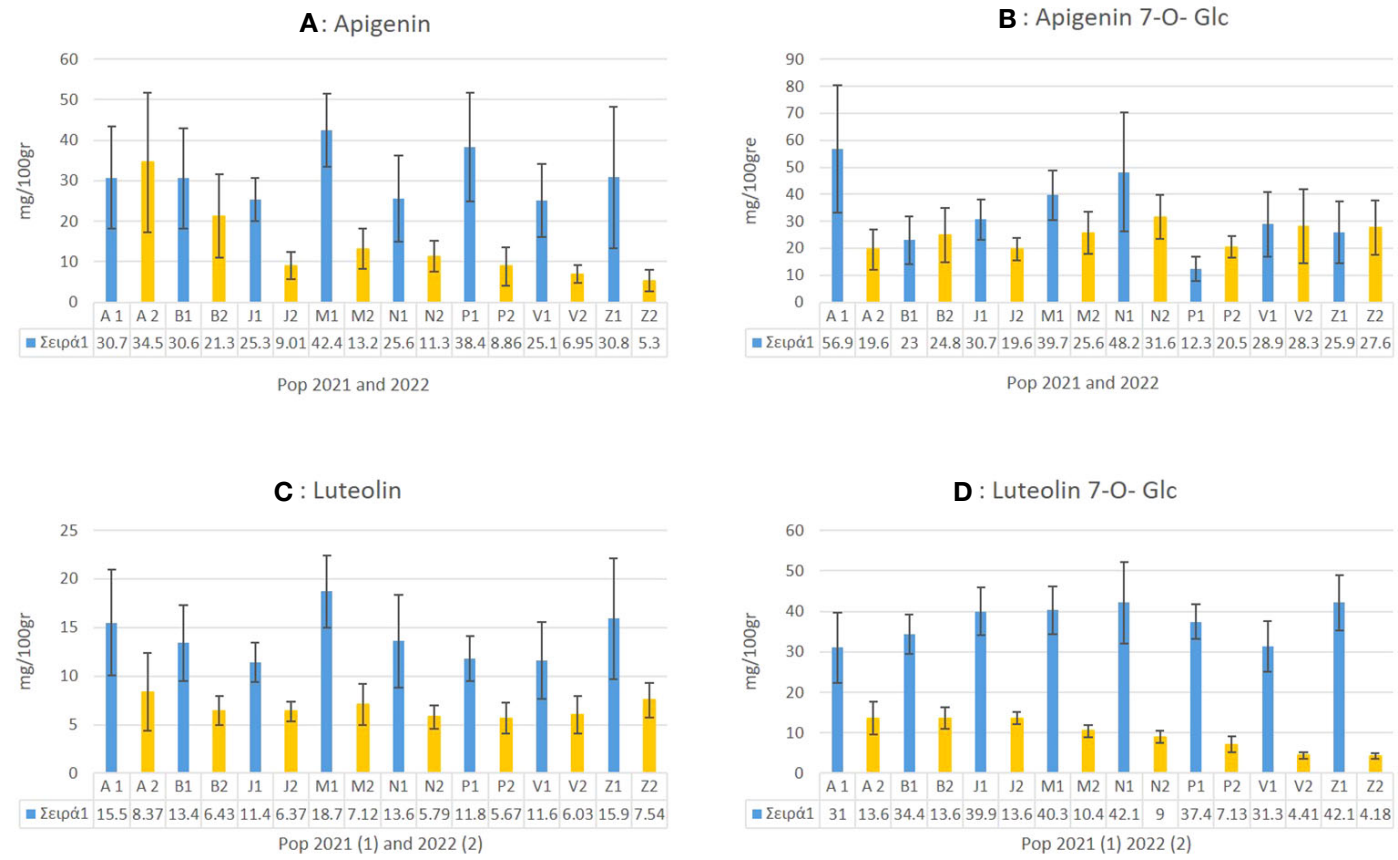
The mean value of apigenin **(A)**, apigenin 7-O-glucoside **(B)**, luteolin **(C)** and luteolin-7-O-glucoside **(D)** and how they differentiate between the eight local populations of *C. alpina* for the years 2021 and 2022 alongside with their calculated SD error bars.

In *C. alpina* the highest values of Ap for 2021 were found for Passo Manghen. A general trend observed was that for most of the accessions, the accumulation of Ap was found to decline significantly during 2022 in comparison with 2021. Agnellezza was the only accession that suppressed the accumulated amounts of 2021 for Ap and the highest producer of this substance in 2022 (**Figure 8A**). For Ap-7-*O*-gluc the highest amounts were found for Agnellezza and Val Nambrone in 2022, while non-significant statistical differences were observed for Bondolo, San Valentino, and Zambana during both years of analysis (**Figure 8B**). The data obtained for luteolin showed that Passo Manghen, closely followed by Agnelezza and Zambana, accumulated the most significant amounts of the flavone during 2021. In the second-year analysis, Agnelezza, Passo Manghen, and Zambana were again the higher producers (13.6 mg/100gr), with the remaining accessions showing no significant differences (**Figure 8C**). In the case of Lu-7-*O*-gluc, Val Nambrone and Juribello showed the highest amounts in 2021, closely followed by Juribello, Passo Manghen, and Peller. In 2022, Agnelezza, Bondolo, and Juribello accumulated the highest values of the flavone (**Figure 8D**).

#### 3.2.4 Correlation analysis

**Figure 9:**
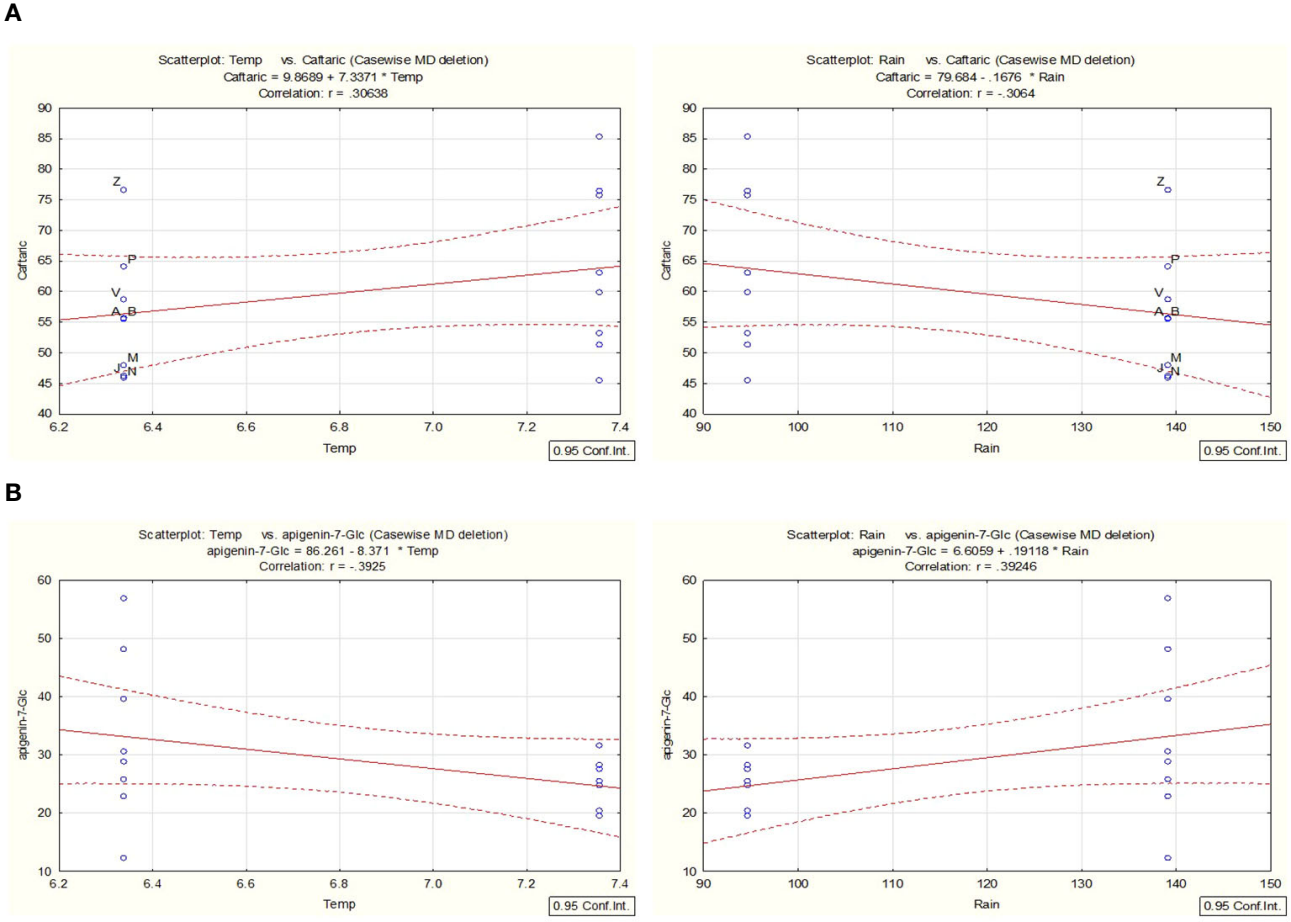
Utilizing STATISTICA, a correlation analysis was conducted to examine the relationship between environmental variables such as temperature and precipitation. The analysis focused on the metabolites caftaric acid **(A)** and apigenin-7-O-glucoside **(B)** identified in *C. alpina* samples collected in both 2021 and 2022. Significance was established at a probability threshold of less than 0.05.

In line with the work presented and to better understand the chemical variability within the years 2021 and 2022, a correlation analysis for selected environmental factors, such as temperature (temp) and rainfall (water availability) (**Supplementary Table S5**), was conducted with the support of STATISTICA for the most prevalent metabolites. For a probability rate < 0.05, the accumulation of caftaric acid was linked with changes that were observed in the parameters of rainfall/water availability and temperature over the two years of cultivation (**Figure 9A**). For example, the overall production of caftaric is positively correlated with a significant decrease in rainfall, which could result in temporary drought stress observed in 2022, while negatively correlated with an overall increase in the mean value of temperature during the same year. The increase observed in the STL content during the second year can also be attributed to changes in the environmental conditions in the area. Cultivar and date of harvest can influence the total STL concentration of chicory (*C. intybus*, *Asteraceae*) leaves, as has been described by Foster et al., 2011 due to changes in the rainfall and temperatures. Thus, similar effects can be proposed for *C. alpina*.

In contrast, correlation analysis proved that the reduction of Ap-7-*O*-gluc during the second year was positively correlated with the decrease in rainfall and was negatively affected by high temperatures (**Figure 9B**).

#### 3.2.5 STL’s and caffeic acid derivatives canonical correspondence analysis

**Figure 10:**
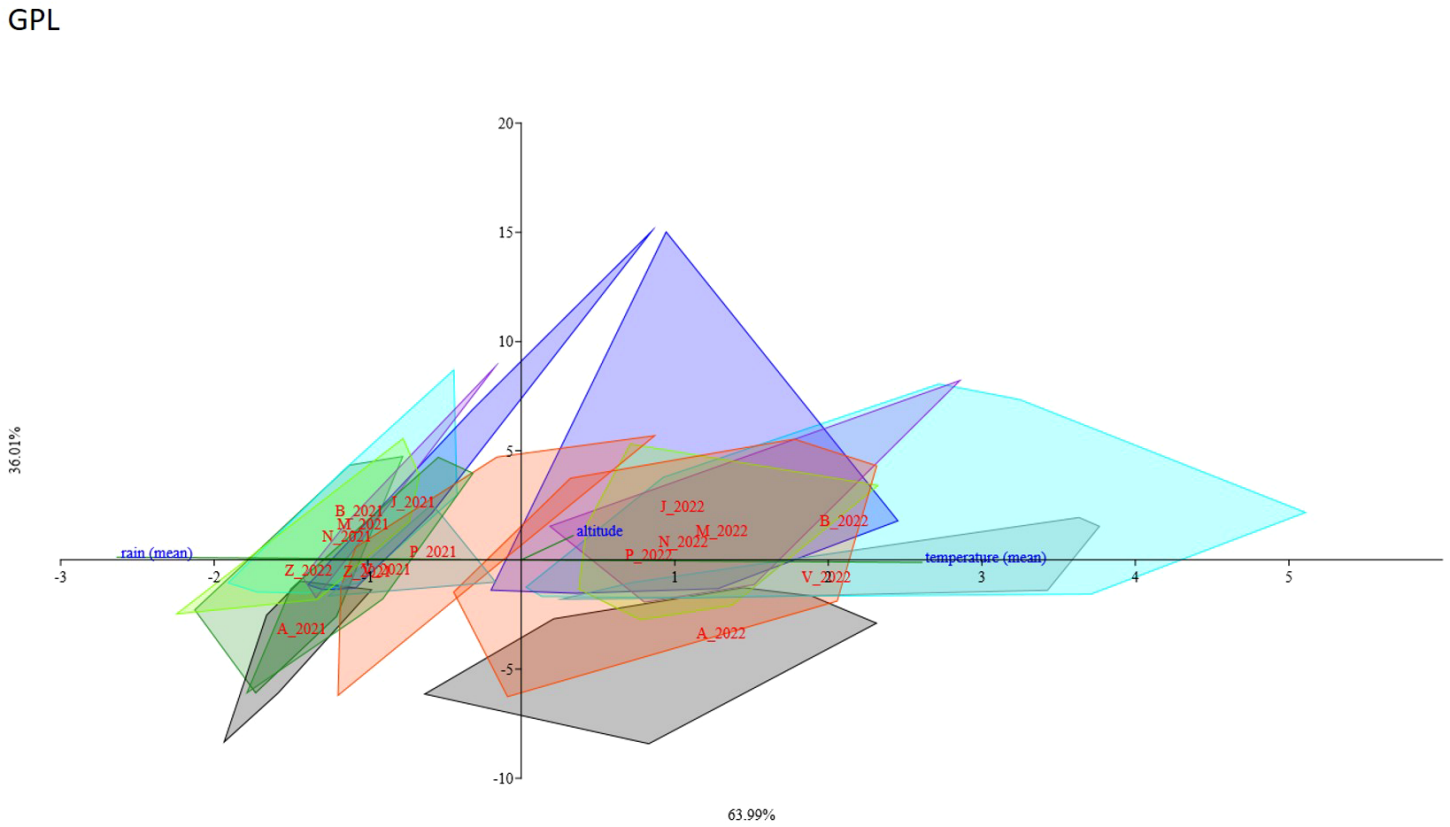
Canonical Correspondence Analysis showed the influence of different environmental conditions (temperature, rain, and altitude) and genotyping data of each population on the accumulation of GPL. The ordination axes were derived as linear combinations of the environmental variables for the years 2021 and 2022, respectively, while each population was identified by a unique color and an initialism: Agnellezza (black/A_2021-A_2022), Bondolo (aqua/B_2021-B_2022), Juribello (blue/J_2021-J2022), Manghen (blueviolet/M_2021-M_2022), Val Nambrone (chartreuse/N_2021-N_2022), Peller (orangered/P_2021-P_2022), San Valentino (cadetblue/V_2021-V2022), and Zambana (forestgreen/Z_2021-Z_2022). Zambana individuals sampled in 2021 and 2022 grouped together, meaning that they are less influenced by water scarcity and environmental differentiation than the rest of the accessions regarding the accumulation of GPL.

A CCA analysis was performed to evaluate the potential influence of the analyzed genotypes *of C. alpina* on different metabolites. The results show that for certain genotypes, climate conditions strongly influence the accumulation of a specific chemical substance. The axis variance was different for the diverse STLs and caffeic acid derivatives: 3.5 DCQ (axis1 94.44%, axis2 5.56%), ChA (axis1 70.87%, axis2 29.13%), GPL (axis1 63.99%, axis2 36.01%) (**Figure 10**), and CGA (axis1 95.66%, axis2 4.341%), respectively. For all compounds, most of the populations sampled in 2021 are clustering in a group and most of the 2022 populations are in a separate group. The 2021 group seems to be positively correlated with water availability (i.e., rainfall) and the 2022 group seems to be negatively correlated with the temperature increase. These results were in accordance with correlation analysis results. Different results were found for Zambana and Agnelezza 2021 and 2022 populations. Zambana individuals sampled in 2021 and 2022 grouped together for GPL and ChA compounds, while on the contrary 2022 Zambana individuals clustered alone for 3.5 DCQ and CGA and seem to be more affected by water availability. Agnelezza individuals sampled in 2021 and 2022 grouped together for all compounds except for GPL where the 2022 group followed the other 2022 populations clustering trend (**Supplementary Figures 3SA–C**).

The results found in the CCA analysis support the trend assessed in the correlation analysis but highlight the influence of the genetic component in the accumulation of metabolites for Agnelezza and Zambana populations. In fact, the first seems to be less influenced by climate change effects while the second seems to be more sensitive towards environmental factors.

## 4 Discussion

Studies on ethnoveterinary plants and the exploration of their antiparasitic effects most probably lead to the identification of promising active compounds not only for veterinary medicine but also for human health (Valente et al., 2021). *C. alpina* is a tall alpine herb that accumulates phytochemicals with demonstrated antiparasitic activity (Poulopoulou et al., 2022), while recent studies have also noted its strong antioxidant response and its potential as a treatment for metabolism-related diseases (Zheleva-Dimitrova et al., 2023). These characteristics render the species an attractive candidate for integration into the pharmaceutical and agricultural industries, warranting further research.

To intensify efforts in domestication and establish a plant breeding program, a thorough evaluation of the genetic diversity of available *C. alpina* germplasm is essential. However, despite its close relation to the *Cichorium* genus, only 30% of tested chicory SSR markers were transferable within the genetic characterization of the current *C. alpina* collection. This outcome could have been expected as similar studies have found that marker pairs effective in cultivated varieties do not always amplify DNA from wild relatives (Wang et al., 2013). Even between species of the same genus, such as *C. endivia* and *C. intybus*, which can interbreed, the successful transfer of microsatellites was less than 50% (Ghedina et al., 2015). These findings highlight/support the high species specificity of these markers, making SSRs a useful tool to study population genetics but also necessitating the confirmation of their transferability or the need for establishing new markers.

The level of heterozygosity (average He) observed in the SSR analysis for the species indicates a high level of cross-pollination, which is expected in natural populations. During a genetic analysis performed for a wild germplasm collection of *Hieracium pilosella* L. (Asteraceae) isolated from ten different locations within the Trentino region (Italy), high levels of polymorphism were detected (Zini and Komjanc, 2006), while in a more recent study during the development of microsatellite locus for *T. cinerariifolium* (Trevir.) Sch.Bip., the observed (Ho) and expected (He) heterozygosity depicted similar values as the ones found for *C. alpina* (Varga et al., 2022). Moreover, the same SSR markers (M1.2, M7.19, M5.14, M4.11b, M4.10a, M2.6, and M1.3), which reveal a medium- to high-level variability within the accessions of *C. alpina*, confirmed restricted gene flow between inbred lines of *C. intybus* (Ghedina et al., 2015). Finally, Mitch and coworkers, noted similar trends within *C. alpina* populations originating from isolated sites in central and northern Europe, with the support of AFLP markers (Michl et al., 2010).

Previous studies support that the genetic structure of the species appears to be closely related to topography and influenced by the level of isolation characteristic of mountainous ecosystems (Michl et al., 2007). In our study, medium to high genetic diversity levels were observed within the eight local *C. alpina* populations. The accession at Passo Manghen, located at the highest elevation (2048 m a.l.t), and San Valentino (1850 m a.l.t) had the lowest Shannon Index value (0.45), followed by Juribello (0.54), originating from the borderlines of the region. STRUCTURE analysis indicated that most of these populations belong to genetic cluster B (marked with green in **Figure 2A**), likely due to their isolation resulting from altitude and/or geographic position (Cisternas-Fuentes and Koski, 2023). In contrast, the highest genetic variability (0.77) was observed in Agnellezza, the population with the lowest elevation level (1503 m), followed closely by Bondolo (0.75) and Peller (0.72), two populations originating from the western part of the region, neighboring the collection sites of Val Nambrone and Zambana. Most of these populations seem to have two common ancestors (Cluster A: blue and C: red), possibly due to their geographic position. Only Agnelezza showed a mix of all three assessed genetic clusters, likely due to its low altitude and proximity to populations on the west side of the river, which separate the eastern from the western area. These conditions make Agnelezza a good candidate for unintentional natural “pre-breeding”. Considering the results, a co-effect between the level of isolation and altitude among the three predicted genetic groups is apparent. Similar observations were described by Stachurska-Swakoń et al. (2011) using AFLP markers. The authors were able to identify that populations originating from higher altitudes in western Carpathians (Poland) had lower genetic variation (Nei and Shannon’s coefficients), compared to accessions collected from lower altitudes in Scandinavia or central Europe. To gain a more global overview of the genetic diversity within the species the here established genetic tools shall be employed to accessions spanning the whole area of distribution, e.g., to similar collections as used in Michl et al. (2007).

The genetic and chemical diversity observed within a species or accession offers valuable information not only about the population structure but also aids in the selection of superior genotypes for plant breeding programs. Hence, in the present study, we examined the chemo-genetic diversity of the samples based on their genetic background (genotype) and chemical composition (chemotype). Chemical diversity does not only occur between plant families, but also within plant genera, plant species, and individual plants of a population (Bączek et al., 2015). Moreover, different species can be defined by the composition of specific metabolites belonging to one specific chemical class, i.e., sesquiterpenes in Asteraceae (Koutsaviti et al., 2022), cannabinoids and terpenoids in cannabis (Jin et al., 2020), and major aromatic substances (like monoterpenes or terpenes) in Lamiaceae (Ramos Da Silva et al., 2021). The most abundant chemical metabolites identified in *C. alpina* include ChA, GPL, CGA, 3.5 DCQ, and caftaric acid, as previously reported in the literature (Fusani and Zidorn, 2010; Zheleva-Dimitrova et al., 2023). Flavones, such as Ap-7-*O*-gluc, Ap, Lu-7-*O*-gluc, and Lu, characteristic for *Asteraceae*, are also present. These metabolites are suspected to possess the main antiparasitic, antioxidant, and AH properties (Poulopoulou et al., 2022., Zheleva-Dimitrova et al., 2023., Ocampos et al., 2023). *C. alpina* remains a species that has received limited investigated attention and as a result, there were few reports to compare the distribution of its metabolites with. According to sources, the concentrations of the main metabolites retrieved were partially in line with previous work by Fusani and Zidorn (2010) and Zheleva-Dimitrova et al. (2023), despite the differences in the extraction and analytical methods used. For instance, ChA was the most abundant compound closely followed by GPL and CGA.

The results of the chemical analysis further confirmed a high variability within and between the accessions, which also indicated significant seasonal variations. Similar studies have observed changes in the total STL and phenolic content of *Cynara cardunculus* L. (artichoke, *Asteraceae*) across different harvesting seasons, suggesting a diverse response closely associated with the cultivar itself (Borgognone et al., 2016). Seasonal patterns between years often correspond to unusual climatic conditions, while alterations in the composition and concentration of STLs and caffeic acid derivatives in *Asteraceae* species can significantly affect forage bitterness (Foster et al., 2011). Overall, the chemical diversity observed in wild MAPs undergoing cultivation trials is substantial and can be attributed to variations in cultivation practices, environmental conditions, abiotic or biotic stresses, and the high level of biodiversity that distinguishes this group of plants (Lubbe and Verpoorte, 2011). The differentiation in the concentration of STLs and caffeic acid derivatives could be attributed to influences by genotype and environmental conditions since the material was growing and harvested from an unirrigated field at Monte Bondone (1503 m of altitude). Furthermore, significant variability in the metabolomic content was also observed within samples from the same accession. For instance, in the Peller population, among the twelve selected plant specimens, it was observed that the fourth plant accumulated 104 mg of GPL, whereas the eleventh plant exhibited twice the amount, specifically 214 mg per 100 grams (**Supplementary Figure 2S**).

In the case of *C. alpina*, the significant increase observed in the accumulation of STLs and caffeic acid derivatives during the second year of evaluation could have triggered, in synergy with other environmental factors, a decline in chemical substances such as polyphenols or flavones. For instance, a preferential increase in the precursor flux of one pathway (caffeic derivatives) might indirectly deplete other closely related pathways that share the same precursors (i.e., flavonoids; Boncan et al., 2020). This observation could explain the decline in major phenolic compounds in contrast to the increase in certain chlorogenic or caffeic acid derivatives.

Furthermore, external abiotic or biotic factors can affect plants and cause changes in their gene expression. In the case of phenolic compounds, it is well-established that within the first twelve hours of observations, stressors can significantly increase the expression of numerous genes, which require considerable energy intake (Kilian et al., 2007). However, when the stress parameter (such as drought, heat, cold, or infection) persists for longer periods (< 24 hours), Kilian et al. (2007) found that the expression of a multitude of genes can be significantly reduced. In *Arabidopsis thaliana* roots Xiong et al. (2006), proved that during long periods of drought stress, there is a significant decrease in the activity of phenylalanine ammonia-lyase (PAL), which is the first enzyme and entry point in the biosynthetic pathway of phenylpropanoids and polyphenols in plants. In conclusion, numerous studies reveal that environmental stresses often tend to increase the accumulation of phenolic compounds, but these findings mainly focus on short-term stresses (Król et al., 2014). The mechanism of response to a long-term and continuous stressor might have different effects. According to Król et al. (2014), it seems that the total phenolic content and antioxidant activity of grapevine leaves and roots are significantly reduced under long-term drought stress. This suggests a deceleration in selected elements of secondary metabolism, during extended water scarcity, a survival strategy against harsh environmental conditions (Akula and Ravishankar, 2011).

These observations can account for the disparities in the levels of phenolic compounds accumulated during the first and second years, characterized by a notable decrease in total rainfall and longer periods of dryness which might cause temporary drought stress. The concentration of polyphenolic compounds can be modulated by multiple variables, with considerable scientific evidence suggesting that environmental factors, including UV radiation, temperature fluctuations, water scarcity, and cold exposure, exert substantial effects. (Nenadis et al., 2015; Bautista et al., 2016; Palmieri et al., 2017; Laoué et al., 2022). Therefore, it was difficult to compare in depth the 2021 and 2022 data. According to meteorological data collected by the Autonomous Province of Trento and publicly available under https://meteo.fmach.it/meteo/index.php (**Supplementary Table S5**), there was a significant decrease in rainfall (-32%) and an increase in temperature (+15%) during the first eight months before the harvest during the second year. Taking everything into account, most polyphenolic substances identified for *C. alpina* showed a significant decline in accumulation for 2022. As an example, the reduction in Ap-7-*O*-gluc during the second year of evaluation is positively correlated with a significant decline in rainfall and is adversely affected by high temperatures. In contrast, the increase of caffeic acid and STL derivatives appear to be positively influenced by the same environmental parameters. The impact of environmental variations on chemical content can sometimes be contingent upon the genetic background of each accession. For instance, from the CCA of GPL, it can be observed that the response of the Agnelezza and Zambana populations to climate change conditions is primarily influenced by individual genotypes. This finding provides a promising foundation for initiating a breeding program focused on *C. alpina*, as plants from these populations are likely less influenced by rain availability and water scarcity—essential traits for developing resilient *Cicerbita* varieties in the face of ongoing climate change. To confirm and enhance these results, future breeding activities and marker-assisted selection work should be undertaken.

In general, the challenges associated with the domestication of *C. alpina* primarily arise from the constraints imposed by its native environment (Scartezzini et al., 2012). Furthermore, the accumulation of phytochemical compounds in *C. alpina* appears to be influenced by genetic and environmental factors unique to mountainous ecosystems. The ability to mitigate or control such variations could play a crucial role in overcoming a significant obstacle to utilizing MAPs as potential sources of natural AH drugs, as well as in establishing plant breeding programs. While establishing a plant breeding program for the domestication of the *C. alpina* species, one approach to be considered is the implementation of controlled pollination techniques involving parent plants sourced from distinct regions (e.g., Scandinavia, the Carpathians, and the Alps) adapted to significantly different climate conditions over the evolution. This strategy could help explore the potential of hybridization between geographically isolated taxa, with the objective of expanding the species’ range to more favorable environments. Furthermore, such research will study the chemical composition of the obtained generations and assess potential impacts arising from such hybridization.

## 5 Conclusions

In conclusion, the present study provided comprehensive insights into the genetic structure and chemical composition of *C. alpina* populations collected in the Province of Trentino. The findings indicate a moderate to high level of variability, suggesting limited gene flow between the local populations. Moreover, the analysis revealed the presence of three distinct genetic groups (K) within the studied populations. For the chemical characterization of the species, the primary focus was on STLs, caffeic acid derivatives, and polyphenols. During this analysis, we identified several substances that have previously demonstrated anthelmintic and insecticidal properties (Valente et al., 2021; Ocampos et al., 2023). Notably, ChA, 3.5 DCQ, GPL, CGA, Ap-7-*O*-gluc, Lu-7-*O*-gluc, Ap, and Lu were found to be the most abundant metabolites. However, it should be noted that *C. alpina* remains relatively underexplored, which poses challenges in drawing final conclusions. Significant variability was observed not only within individual samples of the same accessions but also among the eight distinct populations analyzed. As a result, numerous chemotypes and genotypes varying in their phytochemical profile were characterized for their potent AH activity and other antioxidant properties that have emerged (Ocampos et al., 2023; Zheleva-Dimitrova et al., 2023). Further investigations are mandatory to explore these promising plant species on the chemical and genetic level not only to establish breeding programs aimed at domesticating this species but also to get a better understanding of the underlying adaptation process during evolution enabling the plant to grow at various altitudes and environmental conditions.

## Data availability statement

The original contributions presented in the study are included in the article/**Supplementary Material**. Further inquiries can be directed to the corresponding authors.

## Author contributions

SM: Conceptualization, Funding acquisition, Methodology, Project administration, Resources, Supervision, Writing – review & editing. EM: Formal Analysis, Investigation, Methodology, Writing – original draft, Writing – review & editing. LP: Data curation, Formal Analysis, Supervision, Visualization, Writing – review & editing. MS: Methodology, Supervision. DM: Methodology, Supervision. MO: Investigation, Methodology. LD: Visualization. PF: Funding acquisition, Resources, Writing – review & editing. VT: Resources, Writing – review & editing. IP: Funding acquisition, Resources, Writing – review & editing. MG: Funding acquisition, Resources, Writing – review & editing. MH: Resources, Writing – review & editing. BS: Funding acquisition, Resources, Writing – review & editing. HS: Funding acquisition, Resources, Writing – review & editing.
