# Supplementary material for: Assessment of the chemical and genetic variability among accessions of Cicerbita alpina (L.) Wallr., an alpine plant with anthelmintic properties
Source: Front Plant Sci. 2023 Nov 20;14:1269613. doi: 10.3389/fpls.2023.1269613 (PMC10704909; doi:10.3389/fpls.2023.1269613)

**Supplementary Table S1:** 24 SSRs developed for *C. intybus* (Patella et al, 2019) plus ten loci transferred from *Chrysanthemum* (Wang, 2013) were tested. Primer pairs used for the characterization of genetic diversity in the eight populations of *C. alpina* are given below along their allele size, annealing temperature (An. Tem and) and tail used. PCR amplifications were performed according to the given group and as described in section 2.2.

**Supplementary Table S2:** The two most abundant fragments (1 & 2), known as quantifier and qualifier were noted for the following chemical standard (ID) as shown in the table below. The compound-specific instrumental parameters were optimized accordingly during the method development of *C. alpina*. The Declustering potential (DP) and entrance potential (EP) were improved for each of the precursor ions (Q1), while the collision energy (CE) and Collision Cell Exit Potential (CXP) for each of the product ions (Q3).

**Supplementary Table S3:** Calibration parameters for the LC-MS/MS method built for *C. alpina*. The method was validated according to the accepted US Food and Drug Administration (FDA) bio-analytical method validation guide (US Department of Health and Human Services).

**S3a:** Linear dynamic range (LDR), coefficient of determination (R2) limit of quantification (LOQ), limit of detection (LOD) and matrix effect were obtained using solutions of standards and standard addition in *C. alpina* matrix. LOQs were determined as the concentrations at which the quantifier transition produced a signal-to-noise (S/N) ratio greater than 10, according to the literature.

| **No** | **Compound** |  | **Linearity Range** | **CC In solvent** | **R²** | **CC in Matrix** | **R** | **Matrix Effect** | **LOD** |
| --- | --- | --- | --- | --- | --- | --- | --- | --- | --- |
|  |  |  |  |  |  |  |  |  |  |
| 1 | 1_3 DCQ_1 | | 0.005-10 | y = 4,813,029.63x - 217,290.30 | 1.0 | y = 4390751.65x + 83005.89 | 1.0 | 8.6 | 0.0002 |
| 2 | 3_4 DCQ_1 | | 0.01-10 | y = 4,453,944.59x - 283,806.75 | 1.0 | y = 3699773.34x + 43051.57 | 1.0 | 17.2 | 0.0050 |
| 3 | 3_5 DCQ_1 | | 0.005-10 | y = 8,954,702.67x + 165,580.31 | 1.0 | y = 4737751.46x - 246691.29 | 1.0 | 7.0 | 0.0020 |
| 4 | 4_5 DCQ_1 | | 0.005-10 | y = 8,353,170.66x + 274,023.74 | 1.0 | y = 4759429.58x + 38557.41 | 1.0 | 6.4 | 0.0020 |
| 5 | Shikimic acid_1 | | 0.01-10 | y = 713,501.26x + 49,059.29 | 1.0 | y = 1015203.30x + 6784.81 | 1.0 | 14.7 | 0.0050 |
| 6 | Tartaric acid_1 | | 0.01-10 | y = 2,013,795.13x - 178,396.36 | 1.0 | y = 1059910.01x - 110106.18 | 1.0 | 3.2 | 0.0050 |
| 7 | GPL (HCOOH-)_1 | | 0.005-10 | y = 7,227,380.53x + 431,293.69 | 1.0 | y = 1150691.47x - 45359.39 | 1.0 | 7.9 | 0.0020 |
| 8 | Chicoric acid_1 | | 0.005-10 | y = 2,322,678.62x - 89,348.59 | 1.0 | y = 2797225.90x + 746057.12 | 1.0 | 2.4 | 0.0020 |
| 9 | Lactucopicrin_1 | | 0.001-10 | y = 46,398.32x - 160.98 | 1.0 | y = 22538.67x - 19579.85 | 1.0 | 12.7 | 0.0005 |
| 10 | Lactucin_1 | | 0.02-10 | y = 820,075.75x - 20,171.17 | 1.0 | y = 350895.14x + 46603.18 | 1.0 | 27.6 | 0.0100 |
| 11 | CGA_1 |  | 0.0005-10 | y = 8,780,538.60x + 599,934.12 | 0.9 | y = 91566.50x + 18990.71 | 1.0 | 0.9 | 0.0002 |
| 12 | Caftaric acid_1 | | 0.005-10 | y = 1,086,720.37x - 73,630.64 | 1.0 | y = 783949.77x + 6097853.36 | 1.0 | 27.9 | 0.0020 |
| 13 | p-Coumaric acid_1 | | 0.05 - 10 | y = 6,252,296.87x + 475,249.90 | 1.0 | y = 26112.82x + 5189.84 | 1.0 | 31.0 | 0.0200 |
| 14 | Caffeic acid_1 | | 0.1 - 10 | y = 4,048,894.94x + 321,995.97 | 1.0 | y = 3823056.29x + 859374.25 | 1.0 | 5.6 | 0.0500 |
| 15 | Costonulide_1 | | 0.0005-5 | y = 20,385,200.96x + 858,880.94 | 1.0 | y = 25556471.62x + 251125.28 | 1.0 | -14.6 | 0.0002 |
|  |  | |  |  |  |  |  |  |  |
|  |  | |  |  |  |  |  |  |  |

**S3b** Intra-day and inter-day (CV %) and recovery (%)

|  | **Class** | **Compound** | **Intra-day CV (%)** | **Inter-day (CV %)** | **Recovery 1 ppm** | **Recovery 5 ppm** |
| --- | --- | --- | --- | --- | --- | --- |
| 1 | Caffeic acid derivatives | 1_3 DCQ_1 | 7 | 5 | 96.6 | 93.9 |
| 2 |  | 3_4 DCQ_1 | 2 | 3 | 95.2 | 92.0 |
| 3 |  | 1_5-3_5 DCQ_1 | 1 | 2 | 96.2 | 95.7 |
| 4 |  | 4_5 DCQ_1 | 4 | 4 | 93.2 | 95.3 |
| 5 |  | Shikimic acid_1 | 7 | 12 | 90.2 | 94.6 |
| 6 |  | Tartaric acid_1 | 7 | 7 | 92.5 | 95.2 |
| 7 |  | Chicoric acid_1 | 2 | 4 | 91.4 | 92.8 |
| 8 |  | CGA_1 | 15 | 8 | 98.8 | 94.8 |
| 9 |  | Caftaric acid_1 | 9 | 6 | 90.8 | 93.1 |
| 10 |  | p-Coumaric acid_1 | 2 | 4 | 91.0 | 98.7 |
| 11 |  | Caffeic acid_1 | 2 | 3 | 97.8 | 97.6 |
| 12 | STLs | Costonulide_1 | 2 | 11 | 91.1 | 98.4 |
| 13 |  | GPL (HCOOH-)_1 | 2 | 3 | 93.1 | 94.2 |
| 14 |  | Lactucopicrin_1 | 6 | 12 | 93.3 | 93.3 |
| 15 |  | Lactucin_1 | 8 | 5 | 86.5 | 93.3 |

**Supplementary Table S4a:** The mean value of the most significant STLs and Caffeic acid derivatives identified in the *C. alpina*, and how they differentiate among the eight populations (**A: Agnelezza**, **B: Bondolo**, **J: Juribello, M: Manghen, N: Val Nambrone, P: Peller, V: San Valentino, Z: Zambana)** alongside their calculated standard deviation (SD) error for the years 2021 and 2022.


 **S4b:** The mean value of the most significant polyphenols identified in the *C. alpina*, and how they differentiate among the populations for the years 2021 and 2022. 1) Luteolin, 2) Luteolin-7-O-Glc, 3) quercetin-3-O-glu, 4) quercetin-3-O-GAL, 5) chrysoeriol, 6) neo CGA, 7) daphnetin, 8) esculin, 9) apigenin, 10) apigenin-7-O-Glc, 11) rutin

**Supplementary Table S5:** The meteorological data were provided by the Autonomous Province of Trento and are publicly available at the website <https://meteo.fmach.it/meteo/index.php>. The following table presents the collective temperature (Temp) and rainfall (rain) data from 1/1/2020 to 1/8/2022 (the time of our experiment fieldwork).

| **TEMP** | | | | **RAIN** |
| --- | --- | --- | --- | --- |
|  | **Mean** | **Min** | **Max** | **Mean** |
| 1/1/2020 0:00 | 0 | -8.1 | 9.2 | 64 |
| 1/2/2020 0:00 | 1.3 | -7.8 | 14.8 | 64.4 |
| 1/3/2020 0:00 | 0.6 | -8.2 | 14.2 | 81.7 |
| 1/4/2020 0:00 | 6.2 | -7.6 | 15.7 | 68.8 |
| 1/5/2020 0:00 | 9.3 | 0.6 | 18.5 | 80.2 |
| 1/6/2020 0:00 | 11.9 | 3.8 | 21.6 | 85.6 |
| 1/7/2020 0:00 | 14.7 | 7.2 | 25.1 | 84.8 |
| 1/8/2020 0:00 | 15 | 6.1 | 26 | 85.5 |
| 1/9/2020 0:00 | 11.1 | -0.9 | 19.7 | 89.9 |
| 1/10/2020 0:00 | 5 | -2.7 | 17.2 | 89.6 |
| 1/11/2020 0:00 | 3.7 | -6 | 13.8 | 75.9 |
| 1/12/2020 0:00 | -1.5 | -10.8 | 5.6 | 91.9 |
| **SUM 2020** | **7.9875** | **-1.7375** | **18.5625** | **80.19167** |
| 1/1/2021 0:00 | -4.3 | -13.1 | 6.8 | 81.1 |
| 1/2/2021 0:00 | -0.2 | -14.1 | 12.8 | 80.3 |
| 1/3/2021 0:00 | 0.8 | -9.9 | 15.6 | 66.7 |
| 1/4/2021 0:00 | 2.3 | -8.9 | 15.5 | 76.3 |
| 1/5/2021 0:00 | 6.9 | -0.8 | 14.8 | 82.3 |
| 1/6/2021 0:00 | 14.3 | 5.9 | 22.7 | 81.4 |
| 1/7/2021 0:00 | 14.6 | 5.7 | 22.5 | 84.8 |
| 1/8/2021 0:00 | 13.7 | 4 | 24.6 | 84 |
| 1/9/2021 0:00 | 11.5 | 4.2 | 20 | 87.1 |
| 1/10/2021 0:00 | 6 | -0.8 | 13.5 | 78.5 |
| 1/11/2021 0:00 | 2.4 | -10.4 | 13 | 82.9 |
| 1/12/2021 0:00 | 0.1 | -10.4 | 12 | 72.7 |
| **SUM 2021** | **6.0125** | **-3.9** | **16.9125** | **79.84167** |
| 1/1/2022 0:00 | 0.6 | -10.2 | 13.5 | 54.2 |
| 1/2/2022 0:00 | -0.2 | -9 | 10.3 | 66.5 |
| 1/3/2022 0:00 | 0.4 | -10 | 12.9 | 69.7 |
| 1/4/2022 0:00 | 3.8 | -5.6 | 14.7 | 78.9 |
| 1/5/2022 0:00 | 10.6 | 1.5 | 20.1 | 88.5 |
| 1/6/2022 0:00 | 15 | 6.7 | 24 | 80.6 |
| 1/7/2022 0:00 | 17.1 | 8 | 27.4 | 71.9 |
| 1/8/2022 0:00 | 15.3 | 6 | 24.3 | 78.7 |
| **SUM 2022** | **6.0125** | **-3.9** | **16.9125** | **73.625** |

**Supplementary Figure 1S:** Analysis of two-way ANOVA depicted the efficiency of each factor (time, population, and time * population) for each of the major metabolite compounds isolated from *C. alpina* (significant differences are noted for p < 0.05). The following table presents the two way Anova analysis for GPL. A general trend in the chemical evaluation of *C. alpina* would be that the accumulation of each substance increased significantly during the second year of chemical analyses (2022).


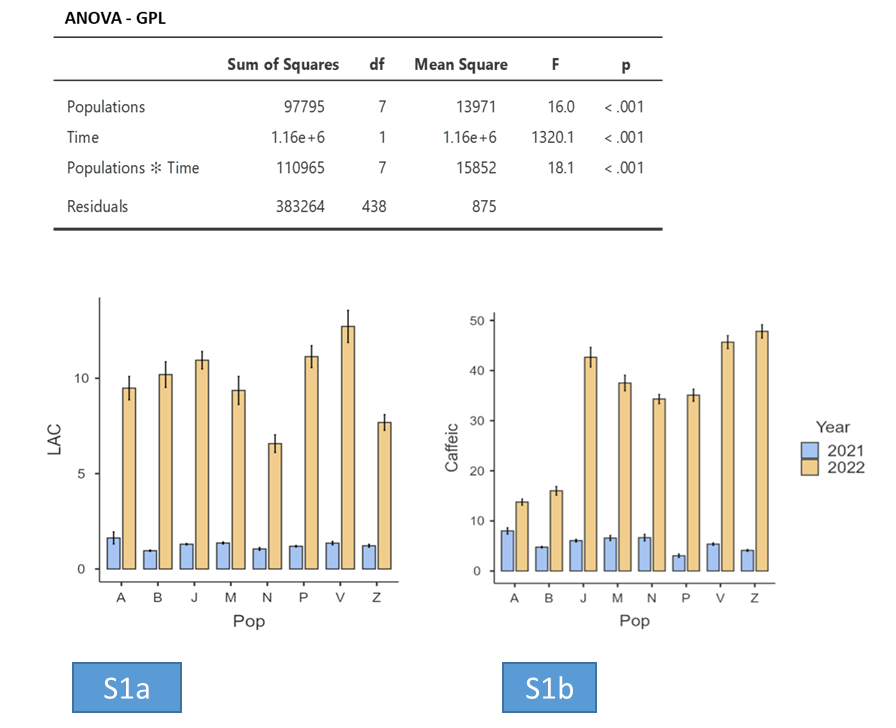


**Supplementary Figure 2S:** In the following chart we can see the accumulation rates of GPL between different specimens within the Peller population alongside their calculated error bars, for the year 2022.

**
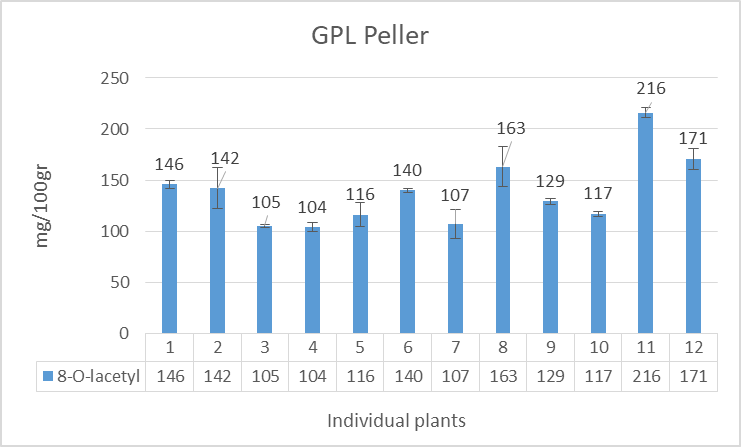
**

**Supplementary Figure 3S:** Using PAST4 software, CCA analysis was performed to evaluate the possible effect of the analyzed *C. alpina* genotypes on various metabolites, such as 3.5 DCQ (3Sa), CGA (3Sb), ChA (3Sc), and GPL (Fig 10) and whether environmental factors such as rain and temperature can influence the accumulation of these substances. For 3.5 DCQ (3Sa) all the *C. alpina* populations sampled in 2021 are clustering in one group and most of the 2022 population in another. The 2021 group seems to be positively correlated with rain availability and the 2022 group seems to be negatively correlated with an increase in mean temperature of the same year. Zambana (Z) 2022 population cluster alone for 3.5 DCQ and seems to be more affected by rain availability than the rest of the accessions. Agnelezza individuals sampled in 2021 and 2022 grouped together and they seemed to be less sensitive to changes in environmental conditions such as temperature and water scarcity. The results confirmed the trend and highlighted the influence of the genetic component in metabolites accumulation especially for Agnelezza (more stable for 3.5 DCQ, CGA, and ChA) and Zambana populations (more sensitive for 3.5 DCQ, CGA, and ChAfor 3.5 DCQ, ChA and CGA). A similar pattern is depicted for CGA (3Sb), while for ChA (3Sc) the overall higher temp during 2022 seems to have prompted an increase in the accumulation of this specific metabolite for all the genotypes.

3Sa


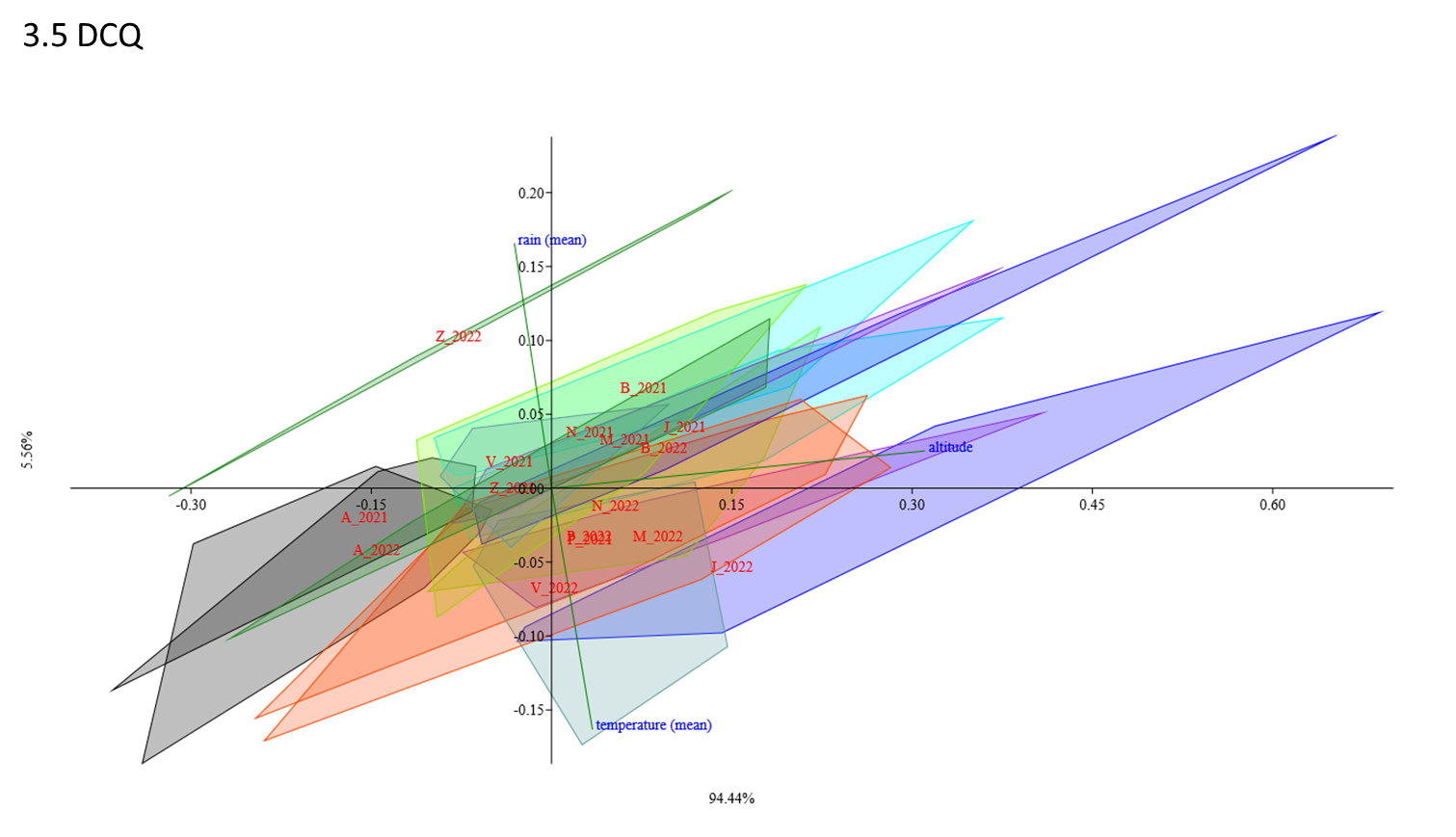


3Sb


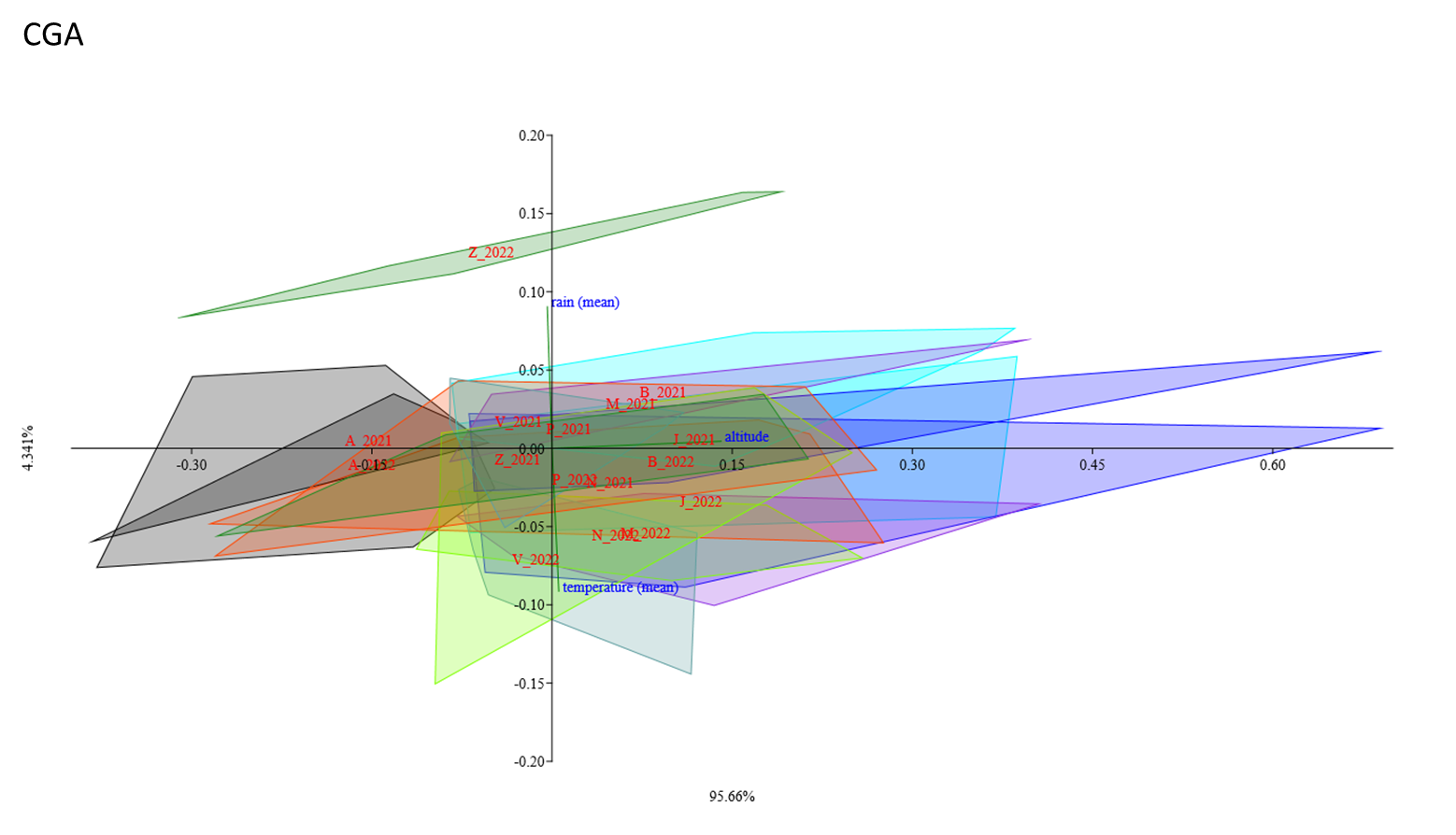


**3Sc**

**ChA**


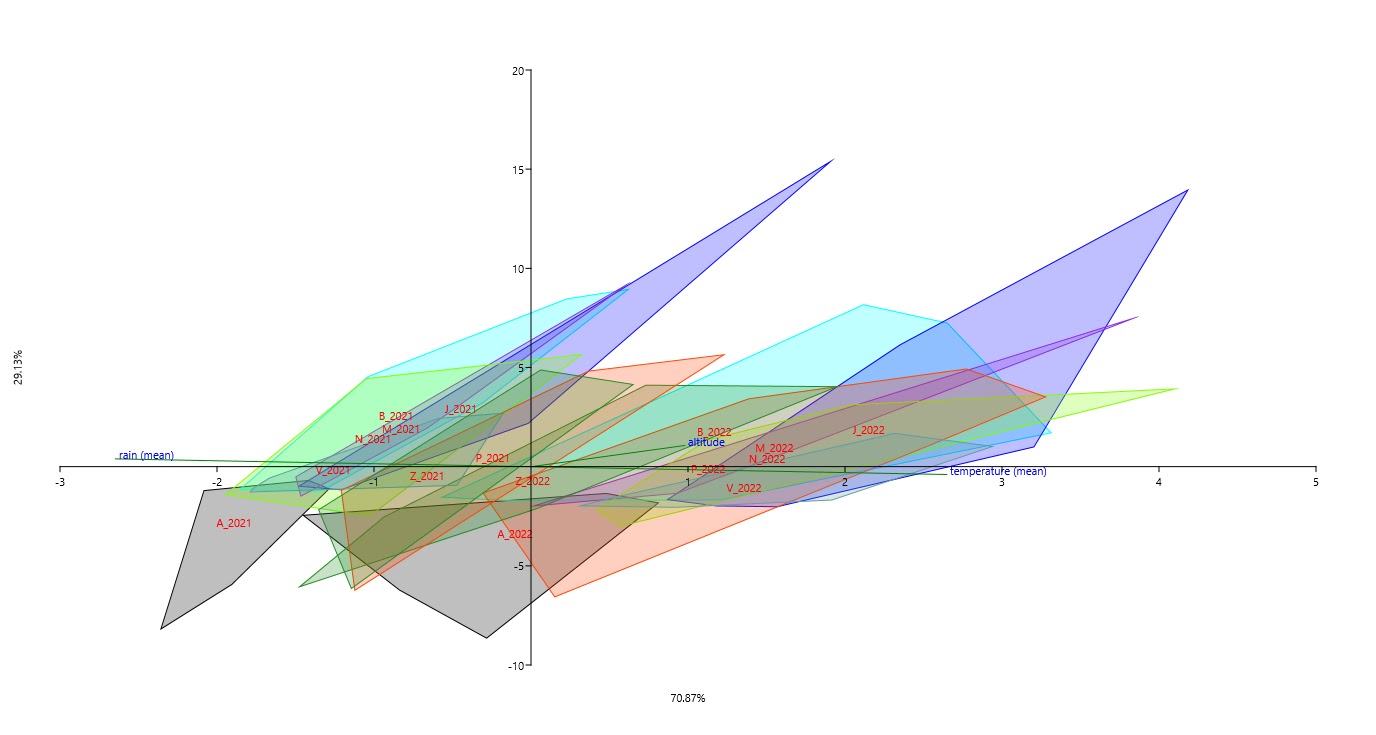

Supplement: Supplementary file 1 [file DataSheet_1.docx]
